# ABC1K2 is involved in stress response and secondary metabolism during seed development in *Arabidopsis thaliana*

**DOI:** 10.1007/s00299-025-03645-0

**Published:** 2025-10-29

**Authors:** Elisa Fasani, Sofia Cardin, Mauro Commisso, Elisa Gecchele, Maria De Benedictis, Gian Pietro Di Sansebastiano, Diana Bellin, Antonella Furini, Giovanni DalCorso

**Affiliations:** 1 Department of Biotechnology, University of Verona, 37134 Verona, Italy; 2 Diamante Srl, 37135 Verona, Italy; 3 Institute of Sciences of Food Production, C.N.R., Unit of Lecce, Lecce, Italy; 4 Department of Biological and Environmental Sciences and Technologies (DiSTeBA), University of Salento, 73100 Lecce, Italy

**Keywords:** ABC1K2, Glucosinolates, Embryogenesis, Seed germination, Abscisic acid, Response to abiotic stress

## Abstract

**Key message:**

**ABC1K2 links stress response and seed development.**

**Abstract:**

The Activity of BC1 complex Kinases (ABC1K) family comprises atypical protein kinases with a conserved ABC1 domain and widespread distribution across life domains. In plants, ABC1Ks are predominantly localised in chloroplasts and mitochondria and have been implicated in energy metabolism, abiotic stress response, and developmental processes. Despite growing evidence of their biochemical activity, the functions of many ABC1Ks remain unclear. This study focuses on *Arabidopsis thaliana* ABC1K2, a plastidial clade member. ABC1K2 is principally localised in seeds; although it shows low expression in vegetative tissues, its transcription is induced by abscisic acid and stress. Functional analysis of *abc1k2* mutants and overexpression lines reveals no major effects on plant development or fertility; however, mutant seeds exhibit increased size, mass, and altered pigmentation, along with reduced ABA levels and modified glucosinolate profiles. Transcriptomic data suggest that ABC1K2 integrates developmental signals with stress responses and secondary metabolism, particularly during seed development and germination. This work highlights a novel role for ABC1K2 in linking hormonal regulation and secondary metabolite biosynthesis, offering new insight into the functional diversification of the ABC1K protein family in plants.

**Supplementary Information:**

The online version contains supplementary material available at 10.1007/s00299-025-03645-0.

## Introduction

The Activity of BC1 complex Kinases (ABC1K) family is a ubiquitous group of proteins that share a highly conserved ABC1 domain (unrelated to the ATP-Binding Cassette – ABC – transporter proteins), whose precise function is still undetermined (Lundquist et al. 2012). The ABC1K proteins are included among the atypical protein kinases (aPK) due to their lack of many features characterising canonical eukaryotic protein kinases; despite this, they share a structurally similar kinase fold domain (Lundquist et al. 2012; Kanev et al. 2019) and have been reported to exhibit kinase activity (Xie et al. 2011; Martinis et al. 2014; Espinoza-Corral and Lundquist 2022).

ABC1Ks have a wide distribution among archaea, eubacteria and eukaryotes; in the latter, they have been isolated from mitochondria, chloroplasts, and the sub-plastidial plastoglobule vesicles (Lundquist et al. 2012). The first ABC1K, COQ8, was discovered and characterised in *Saccharomyces cerevisiae*, where it is required for the biosynthesis of ubiquinone and, therefore, the functioning of the bc1 complex in the respiratory chain (Bousquet et al., 1991; Do et al. 2001). Since then, ABC1K proteins have been described in a variety of organisms, including humans (Lagier-Tourenne et al. 2008) and several plant species such as Arabidopsis, tomato, rice, maize and wheat (Lundquist et al. 2012; Li et al. 2015a; Gao et al. 2022), and more recently in alfalfa and Chinese cabbage (Chen et al. 2024; Ye et al. 2024).

In plants, the ABC1K family has expanded and diversified from yeast COQ8. The Arabidopsis genome harbours 17 ABC1K proteins; similar numbers were also determined in the other investigated species, whereas 44 members were identified in wheat (Lundquist et al. 2012; Li et al. 2015a; Gao et al. 2022; Chen et al. 2024; Ye et al. 2024). These proteins have been assigned to three main clades according to their phylogenetic origin: *i*) ancestral clade, evolved from the ancestral bacterial genomes into the nuclear plant genomes; *ii*) plastidial c., acquired through endosymbiosis from cyanobacteria; and *iii*) mitochondrial c., derived through endosymbiosis from mitochondria progenitors. As a result, plant ABC1Ks have been predicted or determined as localised predominantly in plastids, chloroplast-associated plastoglobules or mitochondria (Gao et al. 2011, 2022; Lundquist et al. 2012; Espinoza-Corral and Lundquist 2022). Given their subcellular localisation, it has been implied that plant ABC1Ks may also be involved in energy metabolism. Indeed, Arabidopsis AtABC1K3 (previously described as ABC1At) partially restores the *coq8* mutation in yeast (Cardazzo et al. 1998), and several studies support the role of plastoglobule-located AtABC1K1 and AtABC1K3 in the maintenance of the plastoquinone pool, in the metabolism of antioxidants, carotenoids and tocopherol and, overall, in the correct functioning of chloroplasts under high light conditions (Lundquist et al. 2013; Martinis et al. 2013, 2014; Pralon et al. 2020). Similarly, rice OsABC1K3 has been reported as involved in chloroplast cyclic electron transport and highlight adaptation (Chen et al. 2023). Also in Arabidopsis, loss of function of the mitochondrial AtABC1K10a resulted in an excessive ROS accumulation under salt stress, likely because of irregularities of the respiratory complex in mitochondria (Qin et al. 2020).

In addition to this role in energy metabolism, ABC1K family expansion has led to the evolution of functions different from those observed in yeast. Several members are affected by stress (Yang et al. 2012a; Li et al. 2015a, b; Wang et al. 2024) and have distinct roles in stress response. For instance, previously mentioned AtABC1K10a modulates the response to salt stress (Qin et al. 2020). In Arabidopsis, plastidial AtABC1K7 (formerly AtSIA) and AtABC1K8 (formerly AtOSA) have been proposed to act in the network connecting abiotic stress response, abscisic acid (ABA) signalling, reactive oxygen species (ROS) production and chloroplast functioning (Jasinski et al. 2008; Yang et al. 2012b; Manara et al. 2014, 2016). Similarly, rice OsABC1-2, wheat TaABC1K3 and TaABC1K6, and cotton GhABC1K2-A05 and GhABC1K12-A07, have been associated with tolerance to abiotic stresses such as dark, drought and salinity (Gao et al. 2012, 2022; Wang et al. 2024). In addition to participating in abiotic stress response, wheat TaABC1K13 (formerly TaABC1) was also found as involved in hypersensitive response to fungal pathogens (Wang et al. 2011, 2013). Furthermore, some ABC1Ks were implicated in a variety of developmental processes. For instance, Arabidopsis AtABC1K1 participates in red light-mediated development by modulating the signalling chain associated with phytochromes (Yang et al. 2016). OsAGSW1, localised in rice chloroplasts, plays a role in the development of vascular bundles and the determination of grain size, the latter by regulating the number of external parenchyma cells and the process of grain filling (Li et al. 2015a, b). More recently, AtABC1K6, another plastoglobule-localised ABC1K kinase, was proposed as needed for a timely transition to the reproductive stage in Arabidopsis (Espinoza-Corral and Lundquist 2022). However, although increasing information has been achieved on ABC1K proteins and their molecular characteristics and biochemical properties have been well studied, the role performed by most of them in plants remains elusive.

Recent research has shown that secondary metabolism plays an essential role in plant communication and response to the surrounding environment and ecosystem (Dixon and Dickinson 2024). Among their functions, secondary metabolites are involved in adaptive processes to environmental stresses, both biotic and abiotic (Erb and Kliebenstein 2020; Khare et al. 2020). In this field, they seem to have some interaction with ABC1K members: for instance, in Arabidopsis loss of function of plastidial AtABC1K7 and AtABC1K8 altered a variety of metabolites, including sinapates (Manara et al. 2015), and similarly, *abc1k1/k3* double mutants showed depleted carotenoid content (Lundquist et al. 2013). One of the fascinating, although poorly described, secondary metabolic pathways in plants is the biosynthesis of glucosinolates, a group of bioactive products derived from amino acids that are found in the order Brassicales (Mitreiter & Gigolashvili 2021). These metabolites have attracted attention as a class of defence compounds against pathogens and insects and because they exhibit various health-promoting properties when assimilated into human nutrition (Traka 2016; Burow and Halkier 2017; Mitreiter and Gigolashvili 2021).

In this paper, we investigate the function of the gene *ABC1K2* (locus *AT5G24970* of the *Arabidopsis thaliana* genome), belonging to the plastidial clade according to the proposed classification of Lundsquit and coworkers (2012). The gene is expressed during embryogenesis, as for the GUS assay, and *ABC1K2* mRNA is barely detectable in leaves, shoots or roots, although transcription is induced by stress and exogenous ABA. An *abc1k2* mutant line has been analysed and compared with overexpressing genotypes. Although the lack of *ABC1K2* does not influence the overall plant fitness, development and reproduction, mutant seeds show a reduced ABA content, increased size and mass, as well as reduced pigmentation and different accumulation of several secondary metabolites, including, interestingly, glucosinolates. A transcriptomic analysis confirms that ABC1K2 participates in the cross-talk between seed development and germination, stress response and secondary metabolism.

## Materials and methods

### Plant material and growth conditions

*Arabidopsis thaliana* (L.) plants, variety Columbia-0 (Col-0), were cultivated in vivo in standard soil (Substrate Tray Superfine #31,883, Gramoflor GmbH & Co. KG, Germany) under growth chamber conditions (16/8 h light/dark photoperiod, 23 °C). For in vitro cultivation, seeds were surface sterilised with 70% ethanol for 1 min, followed by incubation for 15 min in 10% sodium hypochlorite and 0.03% Triton X-100. Seeds were rinsed four times with sterile water and sown on solid MS medium (Murashige and Skoog 1962) supplemented with or without (as described in the text where appropriate) 30 g L^−1^ sucrose. Upon vernalization for 2 days at 4 °C, seeds were kept in vitro with a 16/8 h light/dark photoperiod at 23 °C and 150 µmol m^−2^ s^−1^.

To characterise the function of the ABC1K2 protein (AT5G24970), the T-DNA express collection was analysed (http://signal.salk.edu/cgi-bin/tdnaexpress), and the putative mutant line SAIL_708_G02 was identified; the genotype will be reported from now on as *abc1k2*. Seeds were obtained from the European Arabidopsis Stock Centre and sown on soil; PCR screening to identify homozygous mutant individuals was conducted on genomic DNA according to instructions reported on the website mentioned above.

Overexpressing lines were obtained by cloning the *ABC1K2.3* coding sequence (CDS) under the control of the cauliflower mosaic virus 35S promoter. The *ABC1K2.*3 isoform was chosen since it was the only splicing form resulting from the amplification of the transcript performed on silique cDNA. The *ABC1K2* CDS was amplified by PCR with proofreading AccuPrime™ Pfx DNA Polymerase (Thermo Fisher Scientific, Waltham, MA, USA) from Arabidopsis cDNA, using the specific primers ABC1K2-CDS reported in Table 1. The amplified sequence was ligated into the binary vector pBI121 (Clontech), downstream of the sequence of the CaMV 35S promoter, after excision of the *GUS* sequence by digestion with XbaI and SacI. The construct was introduced into competent *Agrobacterium tumefaciens* (syn. *Rhizobium radiobacter*) strain EHA105 and used to transform the *abc1k2* mutant by the classic floral dip method (Clough and Bent 1998). Transgenic lines were selected in vitro on 50 mg L^−1^ kanamycin MS plates, and resistant plants were identified and transferred to soil for seed collection. Among the lines transferred in vivo, two (from now on, *p35S::ABC1K2*#5 and *p35S::ABC1K2*#7) were chosen after verification of the *ABC1K2* mRNA presence and level by real-time RT-PCR using the specific primers RT_ABC1K2 (Table 1).

**Table 1:** Primers used for DNA amplification

Gene amplified	Forward primer (5’–3’)	Reverse primers (5’–3’)
ABC1K2*-*CDS	TCTAGAATGAGGAATGCCGTTGTTGCGCTC	TTATTGGCTCTTATTGTGATGGA
RT_ABC1K2 (*At5g24970*)	CTCAACAAGACCATCAATCTTA	CATTCCCGGTGCAAGCTTC
GUS	TACACCGACATGTGGAGTGA	CCATACCTGTTCACCGACG
pABC1K2.3	AAGCTTAGAGAACACGTTGCCTGGTG	AGTCCGTTAGATTCACCACTCGGATCC
Fw ABC1K2 ATTB1	GGGGACAAGTTTGTACAAAAAAGCAGGCTTTATGAGGAATGCCGTTGTTG	
*ABC1K2*—stop codon	GGGGACAAGTTTGTACAAAAAAGCAGGCTTTATGAGGAATGCCGTTGTTGCG	GGGGACCACTTTGTACAAGAAAGCTGGGTCTTATTGGCTCTTATTGTG
*ABC1K2* – no stop codon	GGGGACAAGTTTGTACAAAAAAGCAGGCTTTATGAGGAATGCCGTTGTTGCG	GGGGACCACTTTGTACAAGAAAGCTGGGTCTTGGCTCTTATTGTGATGGAG
RFA4 (*At2g21420*)	AGACTCTTCTGCATTGACTGC	CAAGTTGCTACACTGGACAC
EXPA25 (*At5g39300*)	AATGTAGGAGGCGCTGGAG	CATCACTCGTCGTAACCCTG
INVINH1 (*At5g46960*)	ATATCAGGATATTGTGGAAGACT	CTTCCTTGAAATCATCCTCGC
MCM2 (*At1g44900*)	GGCCAAGGAATACGACATTG	CCAAGTGATCAGCCTTTTTGG
HSP (*At5g12020* and *At5g12030*)	CAATCCTCGAAGACATGCTTG	ATCTCATCTCCTTTGATTCCAG
MINI3 (*At1g55600*)	GATGTAGATGAAGATGAAGAGG	TTTGGAGGTGGAGGTTGAGG
IKU2 (*At3g19700*)	TCGCTTGAGATACTTAGA	ATTCCAGAGATTCCAGAA
SHB1 (*At4g25350*)	AAGAATGGTGGAAGACAGAGAT	GAGAAGCAGCAACGATGGTC
ACTIN 2 (*At3g18780*)	GAACTACGAGCTACCTGATG	CTTCCATTCCGATGAGCGAT
UBIQUITIN 10 (*At4g05320*)	AGGACAAGGAAGGTATTCCTC	CTCCTTCTGGATGTTGTAGTC

The information for the utilisation of each primer is detailed in material and methods

### RNA isolation, cDNA synthesis and real-time RT-PCR

Total RNA was extracted from different vegetative plant organs or after specific treatments with the TRIzol Reagent (Thermo Fisher Scientific), following the instructions therein. RNA from immature siliques and seeds was obtained by harvesting non-opened green siliques and following the lithium chloride protocol optimised for sugar-rich samples developed by Vennapusa et al. (2020). Three pools of six plants, from each sample, were used as biological replicates. After DNase treatment on 2 µg of RNA, first-strand cDNA was synthesised by means of the Superscript III Reverse Transcriptase Kit (Thermo Fisher Scientific).

Real-time reverse transcription polymerase chain reaction (RT-PCR) was carried out with the Applied Biosystems QuantStudio 3 (Applied Biosystems, Foster City, CA, USA) using the Platinum SYBR Green qPCR SuperMix UDG (Thermo Fisher Scientific). Each reaction (40 amplification cycles) was performed in triplicate, and melting curves were analysed to ensure the amplification of a single product corresponding to the specific target. Primers for the Real-time PCR analysis are reported in Table 1. Data were normalised using the two endogenous reference genes actin 2 and ubiquitin 10 (Table 1) and analysed using the 2^−ΔΔCT^ method developed by Livak & Schmittgen (2001). The amplification efficiency of each primer pair (*c.* 2) was calculated using the LINREGPCR v.7.5 software (Ramakers et al. 2003).

### Analysis of the *ABC1K2* promoter

Genomic DNA (gDNA) was extracted from the leaves of Col_0 *A. thaliana* plants using the Genomic DNA Extraction Kit (Qiagen, Hilden, Germany), following the procedure therein. Using gDNA as a template, 2044 bps upstream of the start codon of *ABC1K2.3* were amplified with proofreading AccuPrime™ Pfx DNA Polymerase (Thermo Fisher Scientific) and the specific primers for pABC1K2.3 (Table 1). The confirmed sequence was searched bioinformatically for transcription factor binding sites using the Promoter analysis tool of PlantPan 4.0 (https://plantpan.itps.ncku.edu.tw/plantpan4; Chow et al. 2024). The amplified promoter sequence was cloned into a pGEM-T easy vector (Promega, Madison, WI, USA), then modified in the internal HindIII site, replacing the sequence *aagctt* (position − 240 from ATG) with *aaactt* through PCR-mediated mutagenesis; the mutation, confirmed by sequencing, altered no known transcription factor motif. The modified *ABC1K2* promoter was then cloned upstream the *GUS* CDS in the pBI121 vector, replacing the CaMV 35S promoter by cutting with the restriction nucleases HindIII and BamHI. The pBI121-pABC1K2::*GUS* vector was introduced into competent *Agrobacterium* strain EHA105, and wild-type Col_0 *A. thaliana* plants were transformed by classic floral dip, as previously described (Clough and Bent 1998). Seeds were sown on MS plates containing 50 mg L^−1^ kanamycin; resistant individuals were selected, transferred to soil, and seeds were harvested after self-fertilisation. After germination, PCR on genomic DNA was performed with specific primers on the *GUS* gene (Table 1), to confirm the insertion of the transgene in the genome, and copy number was determined by segregation on the T2 generation. Three independent single-insertion lines were analysed in the T3 generation; GUS assay was conducted on plant organs, including leaves, stem, siliques, mature seeds, and plantlets, upon germination.

To test the response to ABA and NaCl treatment, transgenic pABC1K2::*GUS* plantlets were germinated and grown for 1 week in hydroponic culture in Hoagland solution (Hoagland and Arnon 1950), then transferred to the same solution amended with 5 µM ABA or 100 mM NaCl. Plants were collected after 8 h and 48 h, and GUS activity was analysed. For detection of GUS activity, plant material was incubated overnight at 37 °C in the appropriate buffer (100 mM sodium phosphate, pH 7.0, 1 mM EDTA, pH 8.0, 2 mM potassium ferrocyanide, 2 mM potassium ferricyanide, 1% Triton X-100, 500 mg L^−1^ 5-bromo-4-chloro-3-indolyl b-D-glucuronide in DMSO). After the development of the blue colour, chlorophylls were removed by incubating the tissues in 70% ethanol at 70 °C. Plants were examined using a Leica DMRB microscope and an MZ16F stereomicroscope (Leica Microsystems GmbH, Wetzlar, Germany).

Leaves from wild-type plants grown in hydroponics and subjected to the same treatments were collected and used to confirm the expression of *ABC1K2* upon NaCl and ABA treatment by real-time RT-PCR, as described above.

### *GFP*-tagging and vector preparation for transient transformation and protein localisation

In silico prediction of ABC1K2.3 protein topology and localisation was performed using the online tool Protter (https://wlab.ethz.ch/protter/start/) and the database PPDB (http://ppdb.tc.cornell.edu/), respectively. *ABC1K2* CDS, with or without stop codon, was amplified using AccuPrimeTM Pfx DNA Polymerase (Invitrogen) with primers listed in Table 1. The PCR products were purified from the agarose gel using the Gel and PCR Clean-Up System kit (Promega) and cloned into the pDONR221 vector (Thermo Fisher Scientific) following the manufacturer's instructions. The correct entry clones were then recombined in the vector pK7WGF2 for GFP fusion in N-terminus (*GFP::ABC1K2*, with the *ABC1K2* sequence containing the stop codon), in the pK7FWG2 for GFP fusion in C-terminus (*ABC1K2::GFP*, with *ABC1K2* without stop codon) and in the pB7RWG2 for RFP fusion in C-terminus (*ABC1K2::RFP*). The GFP constructs were also co-expressed with the previously described ST-RFP (De Benedictis et al. 2012).

### Transient transformation and confocal laser scanning of tobacco leaf epidermal cells

The constructs were introduced into *A. tumefaciens* strain GV3110 and agroinfiltration was performed in wild-type *Nicotiana tabacum* leaves as previously described (Paris et al. 2010). Single or co-infiltrations of two transgenic *Agrobacterium* strains suspension were performed at the same final optical density. Tissue was observed from 36 to 60 h after infiltration.

MitoTracker Red (Thermo Fisher Scientific) was dissolved in a concentrated stock in DMSO to be diluted to 0.4 μM in an isosmotic solution (9 g L^−1^ NaCl, 18.3 g L^−1^ CaCl_2_·2H_2_O, 0.37 g L^−1^ KCl, 1 g L^−1^ glucose). The dye in the isosmotic solution was infiltrated 30 min before mounting samples on microscope slides, and observations were performed for a variable time of about one hour before renewing samples.

Transiently transformed and stained leaf tissue was examined as previously described (De Caroli et al., 2020) using a confocal laser scanning microscope Zeiss LSM 710 and a ZEN Microscope Software Zeiss AG, Oberkochen, Germany. GFP was detected within the short 505–530 nm wavelength range, assigning the green colour, and RFP within 560–615 nm, assigning the red colour. Excitation wavelengths of 488 and 543 nm were used, respectively. MitoTracker Red fluorescence was detected with the same parameters used for RFP. The laser power was set to a minimum, and appropriate controls were made to ensure no bleed-through from one channel to the other.

### Analysis of seed germination

Seed area was determined by photography analysis by using the image analysis software Fiji (Schindelin et al. 2012). Seed weight was measured on an analytical scale as 100-seed weight after imbibition.

For the germination assay, two stocks of seeds were considered: one freshly harvested (FH, one week after siliques completely dried, self-opening) and one that was kept for 4 months at 20 °C at *ca.* 30% relative humidity (after-ripening, AR). Germination assays were performed at 25 °C in darkness by placing 100 seeds of each genotype in Petri dishes on water-moistened paper (with three replicate plates for each sample). Each germination assay was repeated three times. A seed was considered germinated when the radicle protruded through the teguments, and the process was recorded daily for 8 days.

### Metabolite extraction and sample preparation

Three pools corresponding to 150 mg of mature seeds for both wild-type and *abc1k2* were ground in liquid nitrogen to a fine powder. 90 mg were resuspended in 600 µL of an LC–MS grade solution of methanol:water 80:20 (v/v; Honeywell, Seelze, Germany) including 20 mg L^−1^ butylated hydroxytoluene (BHT). After mixing for 30 s, the tubes were placed on a rotary mixer for 16 h at 4 °C in the dark. The extracts were then sonicated at 40 kHz in a Sonica Ultrasonic Cleaner bath (SOLTEC, Milano, Italy) for 10 min before two rounds of centrifugation at 14,000 g, 15 min each. The hydro-alcoholic extracts were diluted 1:4 (v/v) with MS-grade water (Honeywell), including 0.5 pg µL^−1^ d_6_-abscisic acid (d_6_-ABA; Olchemim, Olomouc, Czech Republic) for abscisic acid quantification, and 1:1000 (v/v) for untargeted metabolomics analysis. The diluted samples were finally filtered with 0.2 µm Minisart RC4 filters (Sartorius, Göttingen, Germany); 5 µL were injected into the Ultra Performance Liquid Chromatography – High Resolution Mass Spectrometer (UPLC-HRMS) for ABA quantification, and 1 µL was used for untargeted metabolomics analysis. d_6_-ABA signal was monitored as an internal standard.

### Abscisic acid and untargeted metabolomics UPLC-MS analysis

The analyses were conducted with an ACQUITY I CLASS UPLC system (Waters, Milford, MA, USA) connected to the Xevo G2-XS qTOF mass spectrometer (Waters), featuring an electrospray ionisation source operating in negative ionisation mode for ABA and untargeted metabolomics analysis. Positive ionisation was employed to assist the identification process and to evaluate the content of sinapoyl choline. Mass Lynx v4.1 software (Waters) was used to control the instruments and to perform preliminary data analysis on the generated raw data files. The chromatographic system exploited a Waters ACQUITY UPLC BEH C18 column (2.1 mm 100 mm, 1.7 m) kept at 30 °C; the mobile phases consisted of 0.1% formic acid in water (A) and acetonitrile (B). Detailed descriptions of run conditions, phase concentrations and parameters, and protocols followed for data processing and metabolite identification are described in Commisso et al. (2019). In detail, the experimental conditions were initially set to 99% A and 1% B, with the following elution profile: 0–1 min, 1% B; 1–10 min, 1–40% B; 10–13.5 min, 40–70% B; 13.5–14.0 min, 70–99% B; 14.0–16.0 min, 99% B; 16.0–16.1 min, 99–1% B (restoring initial conditions). Following the gradient, the system was re-equilibrated with 99% A, and elution was completed after 20 min. The flow rate was maintained at 0.350 mL min^−1^. All samples were stored at 8 °C and subjected to randomisation prior to further processing. A quality control (QC) sample was generated by mixing equal amounts of the extracts to monitor the UPLC-qTOF system's performance throughout the experiment. The QC sample was injected at the beginning, in the middle and at the end of the whole analysis. Additionally, a mixture of authentic commercial standards was prepared, comprising 1 ng µL^−1^ of chlorogenic acid, diadzein, and naringenin, 2 ng µL^−1^ of phenylalanine, and 3 ng µL^−1^ of gallic acid, quercetin, and dehydroartemisininic acid. This mixture (1 µL) was injected following each QC analysis to further assess the system's performance. The ion source settings were as follows: capillary voltage at 0.8 kV, sampling cone voltage at 40 V, source offset voltage at 80 V, source temperature at 120 °C, desolvation temperature at 500 °C, cone gas flow rate at 50 L h^−1^, and desolvation gas flow rate at 1000 L h^−1^. Nitrogen was used for both nebulisation and desolvation, while argon was employed for collision-induced dissociation (CID).

The MS method for untargeted metabolomics analysis was designed to acquire data in continuum mode using two scan functions: in function 1, low energy was disabled, and in function 2, the high energy was set to 35 V. The Xevo G2-XS was set to operate in sensitivity mode for both functions, scanning the mass range of 50–2000 m/z with a scan time of 0.3 s. To ensure accurate mass measurement, a lock mass solution consisting of a 100 pg µL^−1^ leucine-enkephalin solution (Waters) was injected at a flow rate of 10 µL min^−1^, generating a signal of 556.2771 in positive mode and 554.2615 in negative mode. For some samples, the high energy was increased to 45 V to achieve enhanced fragmentation of specific metabolites. Moreover, FAST-DDA analysis was performed in negative mode to better assist metabolite identification by using dual-dynamic collision energies, with the low-mass collision energy ranging from 10 to 40 eV and the high-mass collision energy varying from 20 to 80 eV.

A multiple reaction monitoring (MRM) method was set to record the signals of ABA, d_6_-ABA and abscisic acid glucosyl ester (ABA-GE; Olchemim, Olomouc, Czech Republic). The following transitions were used: 263.13 ⟶ 153.09 at 10 V for ABA, 269.17 ⟶ 159.13 at 10 V for d6-ABA, and 425.1812 ⟶ 153.09 at 18 V for ABA-GE.

### Data processing and metabolite identification

The raw data from the untargeted metabolomics analysis were processed using the Progenesis QI software (Waters) and the default parameters. Tentative identification of metabolites was achieved by performing an automatic search across public databases (MassBank, PlantCyc, Plant Metabolic Network, and Human Metabolome Database), comparing m/z ratios, isotope distributions, and fragmentation patterns, as generated by Progenesis QI. Further identification was carried out using Metlin (https://metlin.scripps.edu) with a mass tolerance of 0.003 Da and by consulting an in-house library of authentic standards. Literature references were also used to corroborate the putative identifications. All identifications were manually validated. The detection of characteristic fragment ions was associated with the presence of specific residues, such as sinapic acid (223.0606, 205.0500, 190.0266, 175.0031, 164.0473, and 149.0268; Shybyray et al. 2025), and benzoic acid (121.0289 and 77.039).

For ABA, d_6_-ABA and ABA-GE quantification, the product ions (153.0813 for ABA and ABA-GE and 159.1286 for d_6_-ABA) were manually extracted using MassLynx v4.1 (Waters).

### RNA isolation, cDNA synthesis and transcriptomic analysis

For transcriptomic analysis, total RNA was extracted from three independent pools of immature seeds for both wild-type and *abc1k2* genotypes, each pool obtained from six plants. Immature siliques were removed from the plants and dipped in liquid nitrogen to promote opening. Valves were removed, and seeds were kept in liquid nitrogen for grinding. RNA was extracted following the lithium chloride protocol optimised for sugar-rich samples (Vennapusa et al. 2020) and was quantified using NanoDrop™ 1000 (Thermo Scientific, Waltham, MA, USA). The RNA quality was evaluated with an Agilent 2100 Bioanalyzer (Agilent Technologies). The samples were submitted to cRNA synthesis and labelling, each starting from 200 ng of total RNA and using the Low Input Quick Amp Labelling Kit, One-Color (Agilent) and Cyanine 3 (Cy3)-CTP fluorescent dye following the instructions of the Agilent technical manual (http://www.agilent.com). Aliquots of Cy3-labelled cRNA (1.65 µg) of each sample were hybridised on one sub-array of the Arabidopsis Gene Expression Microarray, 4 × 44 K (G2519F-021169, Arabidopsis V4, Agilent) for 17 h at 65 °C, according to the manufacturer’s manual. Array hybridisation and washing were performed according to the manufacturer’s manual (One-Color Microarray-Based Gene Expression Analysis-Low Input Quick Amp Labelling-Protocol). Each chip was scanned on an Agilent G2565CA Microarray Scanner System (Agilent) according to the instructions of the Agilent technical manual. Feature intensities were extracted using Agilent Feature Extraction Software 10.5.1.1 (Agilent). The hybridisation data were normalised using the value of the 75th percentile. Differentially expressed transcripts were identified by carrying out transcriptional profile comparisons by t test analysis performed using MeV software (http://mev.tm4.org/#/welcome), setting the following parameters: Welch approximation (variance), alpha (overall threshold p-value): 0.01 and p-value based on t-distribution. Fold change was calculated as the ratio between the average of the three replicates of *abc1k2* and the average of the three replicates of the wild-type. Data are available at the GEO database (accession GSE299982, https://www.ncbi.nlm.nih.gov/geo/query/acc.cgi?acc=GSE299982).

Functional enrichment, by means of GO terms, was assessed using the Functional Annotation tool in DAVID (https://davidbioinformatics.nih.gov/; Sherman et al. 2022). A medium classification stringency was applied, with a p-value threshold of 0.05 and an enrichment score threshold of 1. The association of modulated genes with known metabolic pathways in Arabidopsis was identified using the KEGG Mapper tool in the KEGG database (https://www.genome.jp/kegg/mapper; Kanehisa et al. 2022). To confirm the microarray data, real-time RT-PCR analysis was carried out using cDNA from siliques of wild-type and *abc1k2* plants as previously described. The differentially expressed genes tested and the corresponding primers are listed in Table 1.

### Measurement of stomatal aperture

Entire rosette leaves from 4-week-old wild-type, *abc1k2* mutant and p35S::*ABC1K2*#5 and p35S::*ABC1K2*#7 overexpressing plants were harvested in darkness at the end of the night and then floated in a buffer containing 50 mM KCl, 10 mM MES-KOH (pH 6.15), and 100 µM CaCl_2_ at 22 °C. Stomata were pre-opened under bright-field light (100 µmol m^−2^ s^−1^) for 2.5 h and then incubated for 2.5 h with or without adding 10 µM ABA in the incubation medium. Following these treatments, the leaves were fixed overnight (0.1% glutaraldehyde, 4% paraformaldehyde, 0.1 M sodium phosphate buffer, pH 7.2) and decoloured in an ethanol gradient. Stomata were observed directly using a Leica DM RB microscope (Leica Microsystems GmbH) as previously described (Mustilli et al., 2002). At least 60 stomata were measured for each genotype and condition (two leaves from four different individuals for each treatment). The stomatal aperture was calculated using the Fiji platform (Schindelin et al. 2012) and indicated as a width/length ratio.

### Analysis of ABA-induced leaf senescence

Detached rosette leaves were incubated in water or ABA solution (50 µM) for 4 days under a 12 h photoperiod and light intensity of 100 mmol m^−2^ s^−1^, as previously reported (Jia et al., 2013). Each experiment was performed in triplicate. Photographs were taken each day to evaluate the progression of leaf phenotype. After 4 days, leaves were collected and frozen in liquid nitrogen, ground to a fine powder and used for pigment quantification. Anthocyanin content was determined accordingly to the protocol developed by Nakata & Ohme-Takagi, based on the absorbances at 530 and 657 nm of crude leaf extract (Nakata and Ohme-Takagi 2014). The experiments were conducted in triplicate, considering five leaves for each replicate and genotype.

### Statistical analysis

Data in graphs and histograms are represented as mean ± standard deviation. Statistical analysis was performed using GraphPad Prism 9 (GraphPad Software, Boston, MA, USA). When two genotypes (*i.e.*, wild-type and *abc1k2*) were analysed, statistical significance was evaluated by Student’s t-test; statistically significant differences were marked with asterisks according to the significance level. When more genotypes and/or conditions were considered, one-way ANOVA or two-way ANOVA, as needed, followed by a *post-hoc* Tukey’s multiple comparison test, were used. Details regarding the statistical test applied for each analysis are reported in the figure captions.

## Results

### *ABC1K2* is mainly expressed during embryogenesis and upon abiotic stress conditions

**Fig. 1:**
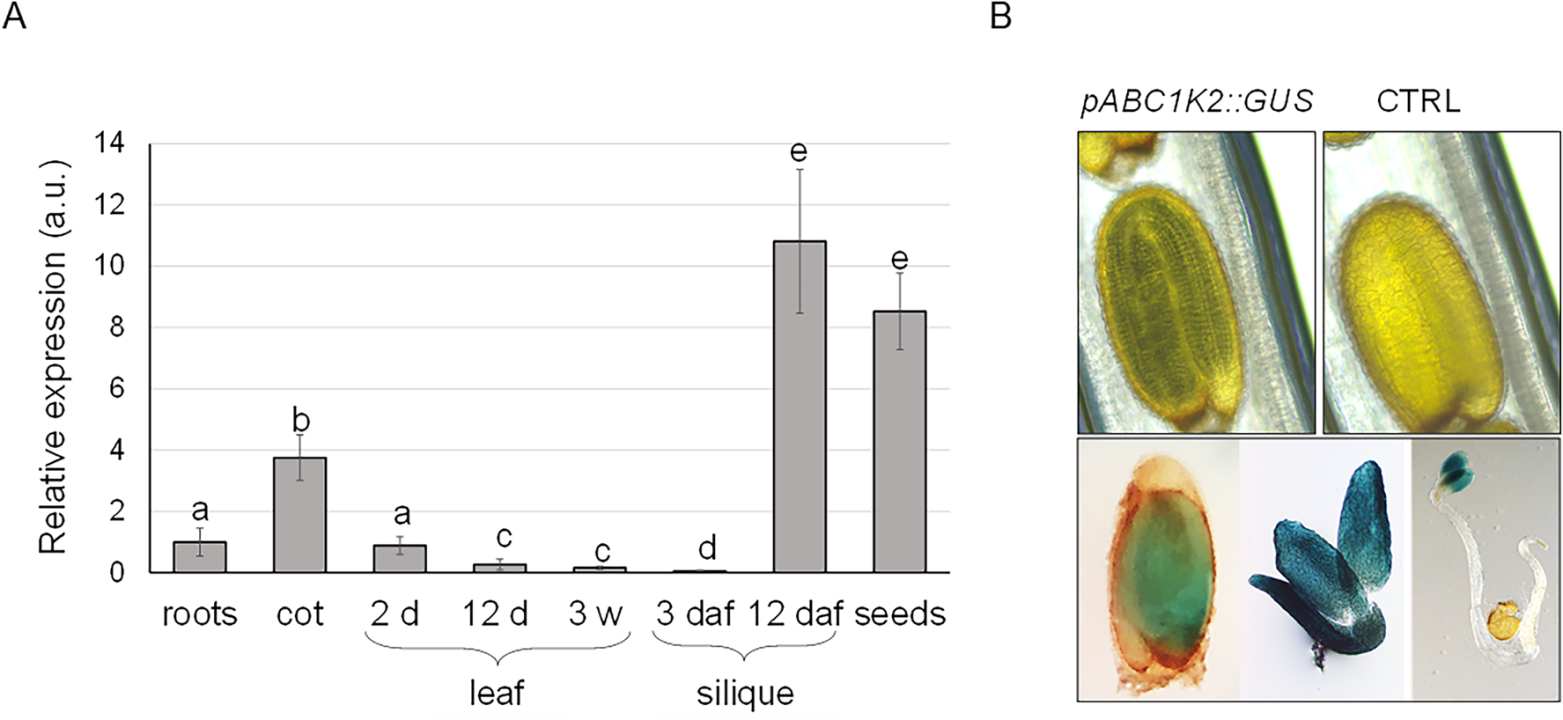
*ABC1K2* expression in different plant organs. **A** Real-time RT-PCR detection of *ABC1K2* transcript in wild-type plant organs. Roots, cotyledons, true leaves of 2 and 12 days and 3-week-old plants, siliques 3 and 12 days after flowering and mature seeds have been separately harvested, and cDNA was synthesised from the purified RNA. Expression in roots has been set to 1 as the reference sample. The analysis was repeated on three biological replicates corresponding to three pools of six plants each, sampled according to the different tissues analysed. Values reported are mean ± standard error of three replicates representative of one pool. Different letters above the bars indicate statistical significance, evaluated by one-way ANOVA followed by a Tukey’s *post-hoc* test (*p* < 0.05). **B** Stereomicroscope images of GUS activity in transgenic individuals harbouring the construct *pABC1K2*::*GUS*. The untransformed seed is shown as a control (CTRL)

To understand the expression profile of *ABC1K2*, in terms of tissue distribution and response to different conditions, a dual approach was considered: transcript levels were determined by real-time RT-PCR, while promoter activity was evaluated by GUS assay. For the latter, the *GUS* gene was cloned under the control of the 2044 bp sequence upstream *ABC1K2.3* start codon. This length was chosen according to previous literature (Hiratsuka et al. 2022). As observed in Fig. 1A, *ABC1K2* is highly expressed in siliques and seeds, as well as more moderately in cotyledons, while it shows lower expression in vegetative tissues. This result prompted us to analyse the tissue localisation of *ABC1K2* in more detail. Analyses of transgenic plants harbouring the pABC1K2*::GUS* construct are reported in Fig. 1B. Confirming the previous real-time RT-PCR results, the *ABC1K2* promoter is mainly active in the embryo. GUS staining is also present in cotyledons, but is not visible in older plantlets or in roots.

To correlate the expression profile with the corresponding *cis-*regulatory elements, the *ABC1K2* promoter sequence was analysed by the PlantPan 4.0 algorithm: the results highlighted the presence of several transcription factor binding sites associated with seed development and germination, as well as ABA signalling, response to salinity and water deficit (Supplementary Fig. 1; Supplementary Table 1). In particular, binding sites for MYC2, MYC3 and MYC4 were identified in the proximal region of the promoter: the three bHLH transcription factors were found as involved in biotic and abiotic stress response and glucosinolate biosynthesis mediated by jasmonic acid (Schweizer et al. 2013; Song et al. 2022), as well as in seed development (Gao et al. 2016).

**Fig. 2:**
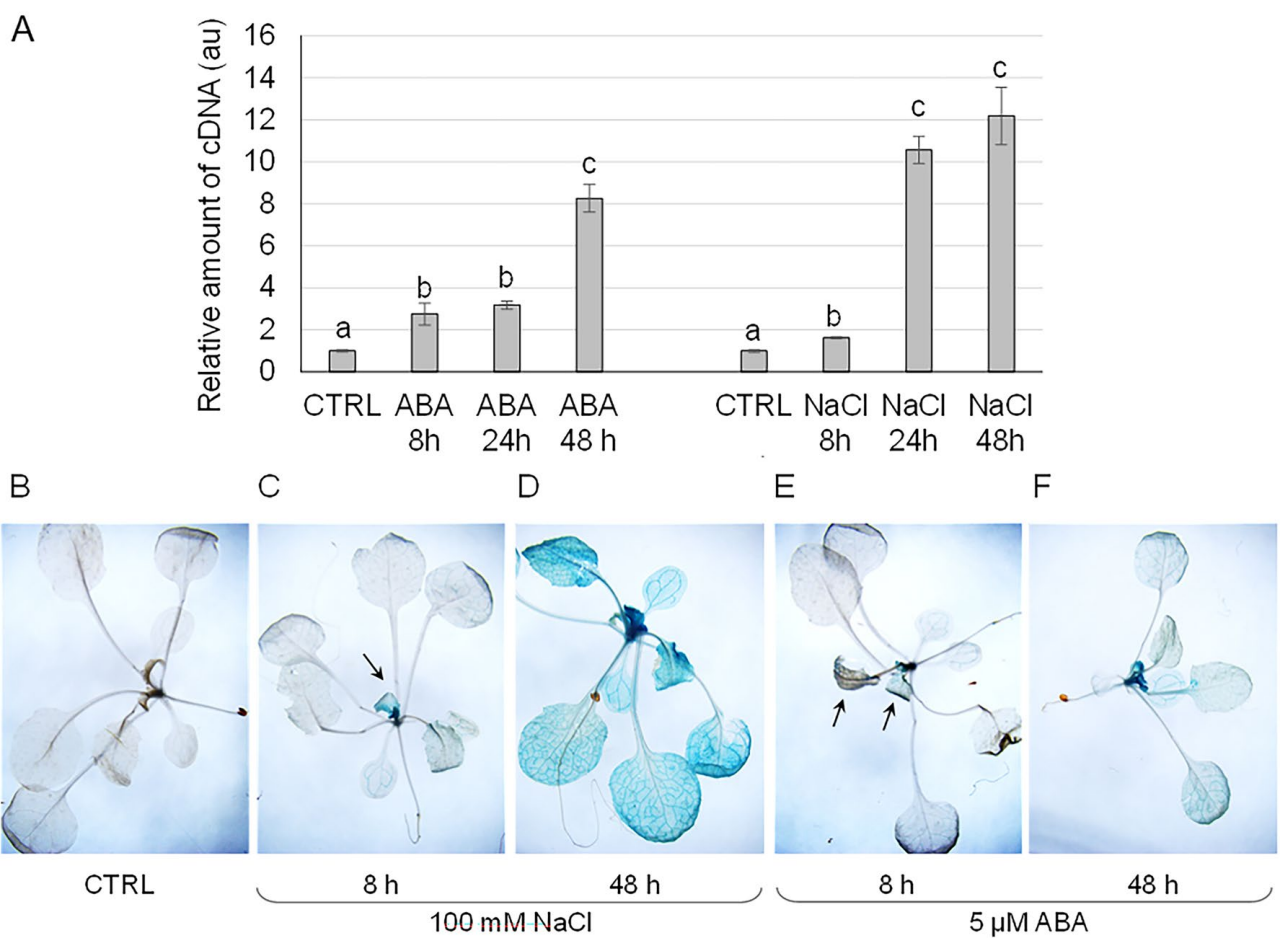
*ABC1K2* expression induction by ABA and NaCl. **A** Real-time RT-PCR quantification of *ABC1K2* transcript in wild-type leaves in control plants and individuals treated with 5 μM ABA or 100 mM NaCl for 8 h, 24 h and 48 h in a hydroponic solution. Expression in the untreated control (CTRL) has been set to 1 as the reference sample. The analysis was repeated on three biological replicates corresponding to three pools of six plants each. Values reported are mean ± standard error of three replicates representative of one pool. Different letters above the bars indicate statistical significance, evaluated by one-way ANOVA followed by a Tukey’s *post-hoc* test (*p* < 0.05); the statistical analysis was performed independently for each treatment. The same treatments were applied to transgenic *pABC1K2::GUS* lines. Representative individuals are reported as follows: **B** transgenic plant maintained in standard hydroponic solution; **C** and **D** transgenic individuals grown in 100 mM NaCl, and harvested after 8 h (**C**) and 48 h (**D**); **E** and **F** transgenic individuals grown in 5 μM ABA and harvested after 8 h (**E**) and 48 h (**F**). Arrows in **C** and **E** indicate blue colouration corresponding to the GUS expression and activity in young leaves

Given the presence of multiple stress-responsive regulatory elements, the putative modulation of *ABC1K2* expression was tested by real-time RT-PCR in wild-type plants challenged with salt stress and ABA treatment. Indeed, *ABC1K2* transcript accumulation was gradually induced in leaves by both treatments with 100 mM NaCl and 5 µM ABA; the result was also confirmed by GUS assay on pABC1K2::*GUS* plants subjected to the same treatments (Fig. 2).

The in silico analysis of ABC1K2, performed by the software Protter, showed that the protein is probably characterised by two transmembrane domains located in the N-terminal portion, separated by *ca*. 80 amino and preceded by a putative, uncharacterised signal peptide (data not shown). Regarding the putative subcellular localisation, no clear prediction emerged from the research against the database PPDB. To understand the localisation of ABC1K2, chimeric constructs fusing the *GFP* to the 3’ end or the 5’ end and the *RFP* CDS to the 3’ end of the *ABC1K2* CDS were transiently expressed in epidermal tobacco leaves, showing a similar subcellular distribution despite a diversified brightness of fluorescence (Supplementary Fig. 2A-D). As shown in Supplementary Fig. 2, GFP-tagged ABC1K2 localisation was not clear. Granulose structures of discrete size could be observed for GFP::ABC1K2 (Supplementary Fig.  2 A) or ABC1K2::RFP (Supplementary Fig. 2B), as well as for ABC1K2::GFP (Supplementary Fig. 2D). Since punctate structures resemble the shape of Golgi dictiosomes or mitochondria, co-localisation of ABC1K2::GFP was performed, using the Trans Golgi network fluorescent protein marker ST-RFP (De Benedictis et al. 2012) and the mitochondria dye, MitoTracker Red. The results show that ABC1K2::GFP co-localises neither with ST-RFP (Supplementary Fig. 2D-F), nor with the mitochondrial marker MitoTracker Red (Supplementary Fig. 2G-I).

### Knock-out and overexpressing lines identification and confirmation

A mutant line for *ABC1K2* (from now on, *abc1k2*) was identified by searching a T-DNA insertion line in the SAIL collection of mutants created in *A. thaliana* Col-0. The insertion site was confirmed by PCR with gene-specific and T-DNA-specific primers, followed by sequencing of the PCR products. The *abc1k2* mutant contains a T-DNA insertion in the eleventh intron of the genomic sequence (Supplementary Fig.  3 A). To confirm that the mutant was s knock-out and thus the T-DNA insertion effectively disrupts gene transcription, total RNA was isolated from wild-type and *abc1k2* siliques, and, after cDNA synthesis, a real-time RT-PCR analysis was performed using gene-specific primers. The study demonstrated that the *abc1k2* mutant showed no residual transcription of the *ABC1K2* gene (Supplementary Fig. 3B). Since only one mutant line has been identified, we created complementing lines by overexpressing the *ABC1K2* CDS, under the control of the CaMV 35S promoter, in the *abc1k2* mutant background. Real-time RT-PCR analysis performed of leaf cDNA confirmed the expression of *ABC1K2* in most lines. Of those, two lines, p35S*::ABC1K2*#5 and p35S*::ABC1K2*#7, were adopted for further experiments (Supplementary Fig.  3 C).

### *ABC1K2* disruption influences seed germination, dormancy release and ABA content in seeds

**Fig. 3:**
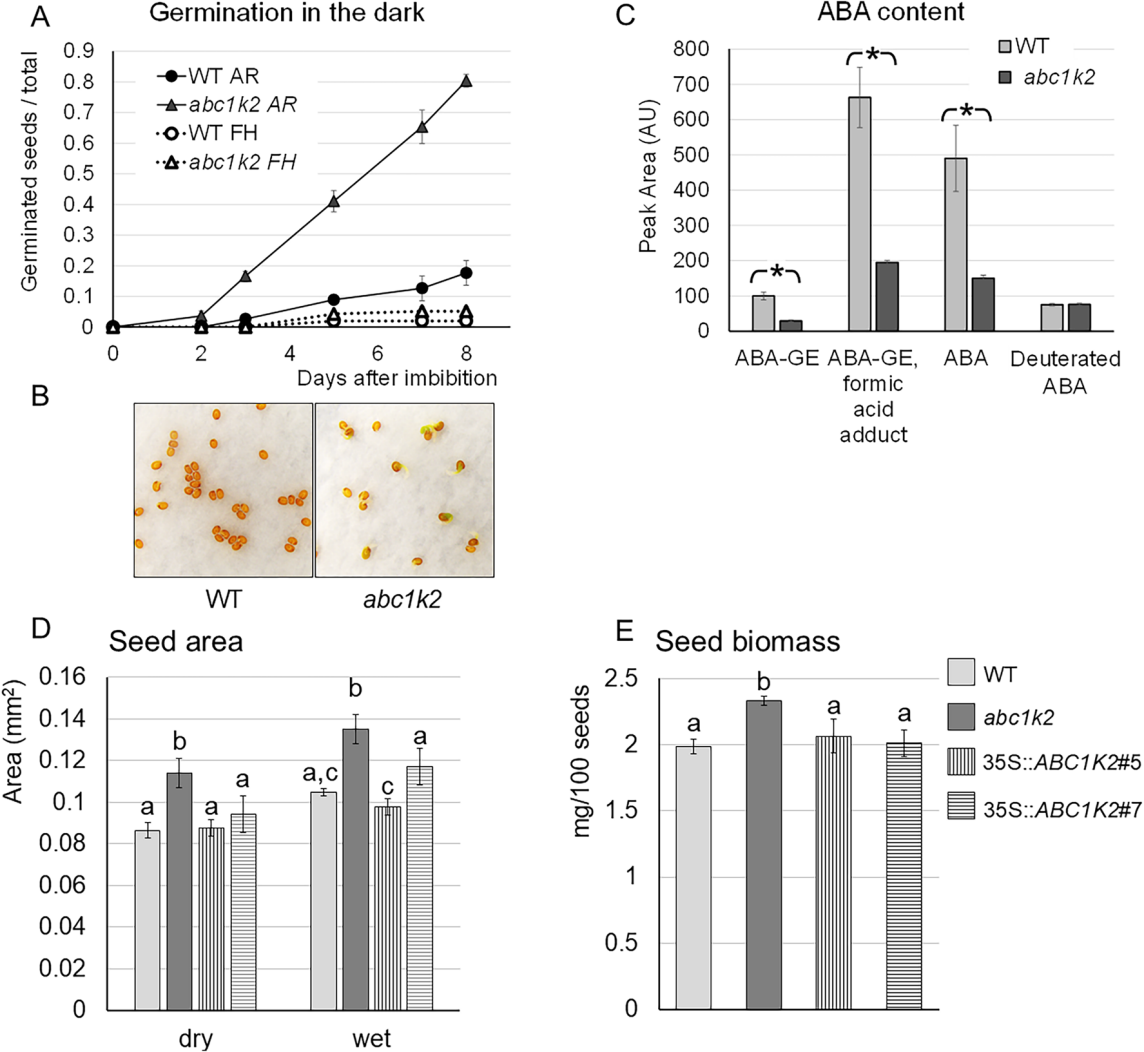
*abc1k2* seeds show a reduced dormancy and ABA content. **A** Germination assay of wild-type and *abc1k2* seeds, where FH indicates freshly harvested seeds, one week after siliques completely dried, and AR indicates after ripening seeds, stored for 2 months at 20 °C and ca. 30% relative humidity. Germination assays were performed at 25 °C in darkness by placing 100 seeds of each genotype in Petri dishes on a humid paper disk. A seed was considered germinated when the radicle emerged from the tegument. Results in the graphs correspond to the mean germination fraction of 100 seeds (± standard deviation) for each of the three Petri for each condition. **B** Representative photography of AR seeds of wild-type and *abc1k2* mutant, taken 8 days after imbibition. **C** ABA quantification by UPLC-MS. ABA-GE: ABA glucosyl ester; ABA-GE formic acid adduct: ABA glucosyl ester conjugated with a formic acid molecule. The adduct molecular species are formed during ionisation in the mass spectrometer and, therefore, are not naturally occurring molecules in the sample. Deuterated ABA was added from the commercial authentic standard (d_6_-ABA) in equal volume to wild-type and *abc1k2* samples as an internal control. Data in the histogram are reported as the mean of three biological repetitions ± standard deviation. Asterisks above the histograms indicate statistical significance, evaluated by Student’s t–test: *p < 0.05. Seed area (**D**) and seed size (**E**) were measured in seeds of wild-type, abc1k2 mutant and complementing lines #5 and #7. Results correspond to the mean ± standard deviation obtained from three samples of 100 seeds for each genotype. Seed biomass has been measured on imbibed seeds. Different letters above the bars indicate statistical significance, evaluated by one-way ANOVA followed by a Tukey’s *post-hoc* test (*p* < 0.05); statistical analysis in A was performed on dry and imbibed seeds separately

Freshly harvested *A. thaliana* seeds are usually dormant and unable to fully germinate on water-soaked paper at 25 °C in darkness (Basbouss-Serhal et al. 2016). Consistently, freshly harvested (FH) wild-type and *abc1k2* seeds poorly germinated under these conditions after 8 days upon imbibition. On the contrary, after 4 months of storage at 20 °C and 30% relative humidity (AR, after ripening), while wild-type seeds still retain dormancy and germination does not proceed over 20%, *abc1k2* seeds show dormancy break, and germinate almost completely within 8 days post-imbibition (Fig. 3A, B). ABA activity in the embryonal tissues influences seed dormancy and germination during embryogenesis (Ali et al. 2021). ABA content was therefore measured in freshly harvested seeds of both wild-type and *abc1k2*. As shown in Fig. 3C, the content of the primary ABA forms is lower in *abc1k2* seeds when compared to the wild-type ones, while deuterated ABA, introduced as an internal reference, was not different between the two genotypes.

A detailed observation of seeds revealed that the loss of ABC1K2 also results in a perturbed control of seed dimensions: *abc1k2* seeds showed increased area and biomass, while the wild-type phenotype was recovered by the overexpression of *ABC1K2* in mutant lines (Fig. 3D, E).

### *ABC1K2* influences ABA-induced leaf senescence and stomatal opening

**Fig. 4:**
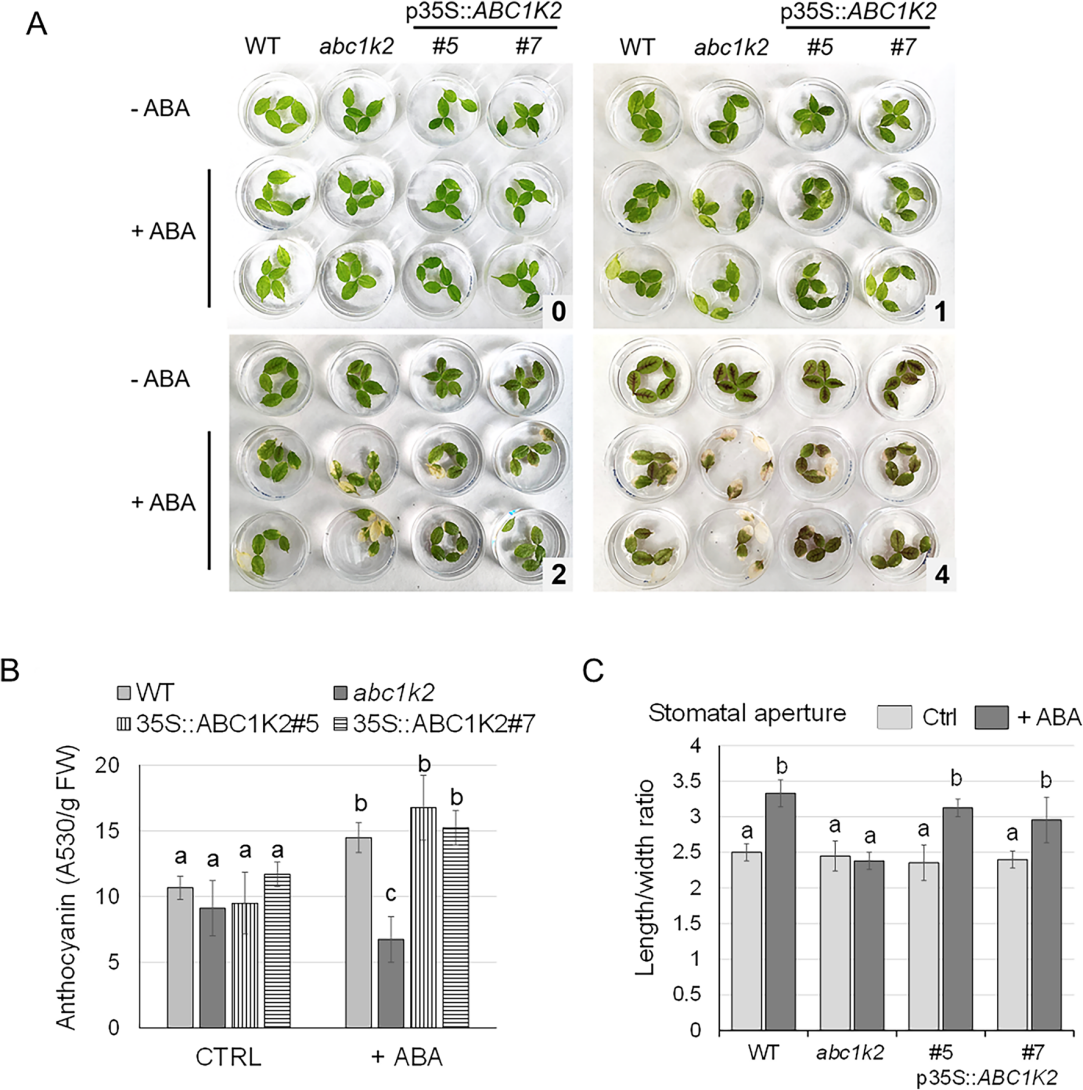
ABC1K2 is involved in ABA signalling in leaves. **A** Leaves detached from WT, *abc1k2* and overexpressing lines were floated on sterile water (– ABA**)** or 50 μM ABA (+ ABA) for 5 days. Image of detached leaves at day 0 (panel 0), and after 1 (panel 1), 2 (panel 2) and 4 days (panel 4). **B** Anthocyanins quantification in floating detached leaves upon ABA treatment conducted in **A**. Leaves picked from Petri dishes displayed in panel **A** were frozen in liquid nitrogen and ground into a fine powder. Anthocyanin contents were analysed as described in M&M, and data reported are mean ± standard deviation of three biological replicates, considering five leaves for each replicate, each genotype. **C:** Stomatal aperture was analysed on detached leaves upon treatment with water (ctrl) or 10 µM ABA (+ ABA) for 2.5 h. Stomatal aperture was determined as the length: width ratio of each stoma. Data reported are relative to 60 stomata analysed for each genotype, represented as mean ± standard deviation. Different letters above the bars indicate statistical significance, evaluated by Welch’s ANOVA followed by a Games–Howell post-hoc test (*p* < 0.05)

Since *ABC1K2* expression is induced by ABA in the leaf tissue, the effect of ABA-induced leaf senescence was investigated on detached leaves of wild-type, *abc1k2* mutant, and p35S::*ABC1K2*#5 and p35S::*ABC1K2*#7 overexpressing plants. Leaves were floated on water or 50 μM ABA for 5 days and senescence was assessed by observing changes in leaf appearance (Fig. 4A). Detached leaves floated on water for 5 days showed no visible yellowing regardless of the genotype. In contrast, those floated on the ABA solution developed chlorotic patches. Interestingly, under this condition, wild-type and overexpressing leaves showed anthocyanin production (Fig. 4B), which has been ascribed to ABA-induced (Shi et al. 2022; Gao et al. 2023). On the other hand, the ABA treatment also induced chlorosis and senescence in *abc1k2*, but detached leaves showed reduced anthocyanin accumulation (Fig. 4A, B).

Due to the role of ABA in stomatal closure control, the effect of *ABC1K2* loss of function in the ABA-mediated stomatal response was investigated by exposing epidermal strips of wild-type, *abc1k2* and overexpressing lines to light in the absence or presence of ABA. Following exposure to light (100–120 μmol m^−2^ s^−1^) in the absence of ABA, the stomatal aperture, measured as length:width ratio, of all genotypes tested was comparable. On the contrary, ABA treatment (10 µM ABA for 2.5 h) induced stomatal closure only in wild-type and overexpressing lines, whereas the stomata closure in *abc1k2* was inhibited (Fig. 4C). The stomatal density and size under normal conditions were not influenced by *abc1k2* knock-out or overexpression (data not shown).

### *abc1k2* mutant shows an altered secondary metabolism in seeds

**Fig. 5:**
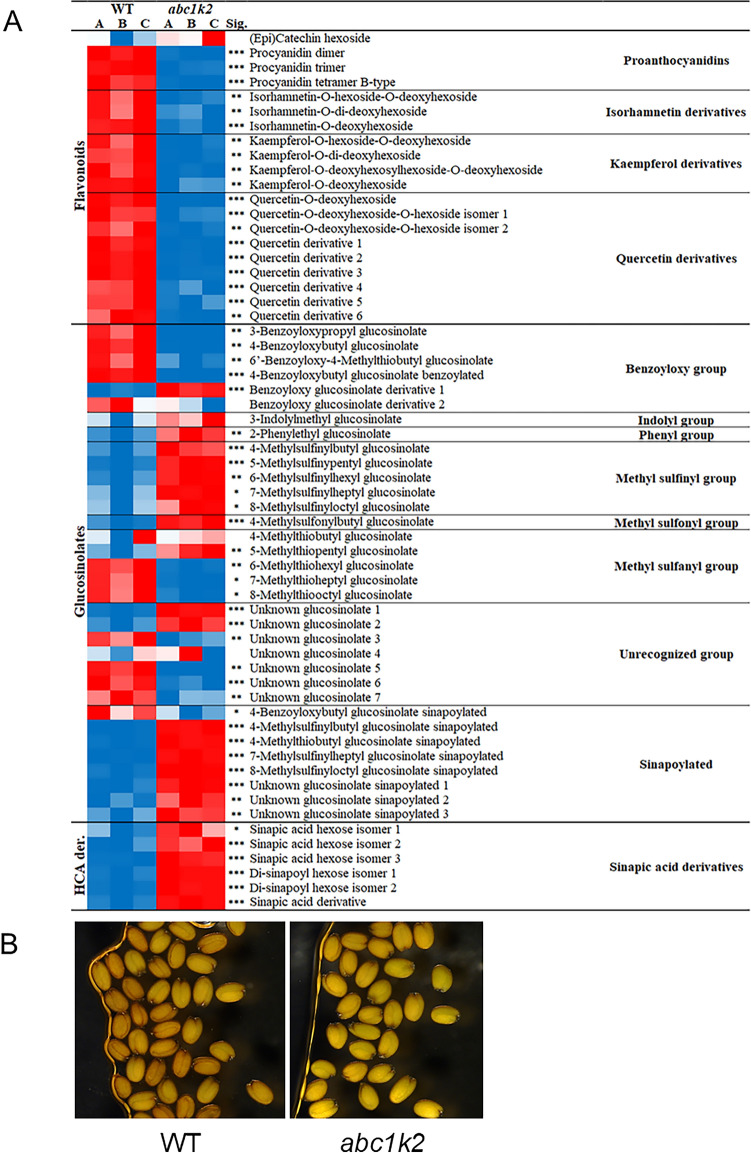
**A** Heat map displaying the different abundances of the main metabolite classes between WT and *abc1k2* seed extracts. The heatmap was generated using the conditional formatting tool in Microsoft Excel (v. 2401), with the red colour set as the maximum value, white as the 50th percentile, and blue as the minimum value (values are reported in Supplementary Table 2). A, B and C indicate the biological replicates corresponding to three seed pools harvested from six plants each pool. HCA der: hydroxycinnamic acid derivatives; Sig. statistical significance (Student’s test); *: *p*-value < 0.05; **:*p*-value < 0.01; ***:*p*-value < 0.001. **B**, picture at the light microscope of WT and abc1k2 imbibed seeds fixed in nail polish on a microscope glass

Harvested mature seeds from wild-type and *abc1k2* were submitted to a comparative analysis of the secondary metabolites by LC-HRMS analysis by an untargeted metabolomics approach in negative ionisation mode. The chromatographic data were processed through Progenesis QI and resulted in a data matrix including 458 m/z features with 83 signals putatively identified and grouped in specific chemical classes (Supplementary Table 2). The specialised metabolome of *A. thaliana* seeds mainly includes glucosinolates, flavonoids and hydroxycinnamic acids, representing 66%, 12% and 1% of the total signal in the wild-type, respectively. In *abc1k2*, the total metabolite content was *ca.* 17% lower (*p*-value < 0.05) than that of the wild-type. However, it is worth mentioning that, despite this reduction, the base peak ion (BPI) chromatograms of wild-type and *abc1k2* samples exhibited similar profiles, allowing for a comparative analysis (Supplementary Fig. 4). Overall, *abc1k2* showed a general reduction in all the classes of flavonoids, an increase in hydroxycinnamic acids and a variable behaviour regarding glucosinolates (Fig. 5A). The heatmap displays all the putatively identified metabolites, systematically grouped according to their respective metabolite classes, and highlights the distinct distribution patterns of each metabolite. Differences were noticeable among the most abundant flavonol glycosides characteristic of *A. thaliana* seeds, which are based on quercetin, kaempferol and isorhamnetin aglycones (Routaboul et al. 2006). The entire biosynthetic pathway downstream dihydrokaempferol seems to be inhibited in *abc1k2*, with quercetin derivatives displaying the highest reduction, especially quercetin-O-deoxyhexoside (Supplementary Table 2). It should be noted that the flavonol glycosides are only reduced in *abc1k2*, but there is no lack of particular compounds, as reported for other mutant lines impaired in flavonol accumulation, such as *tt7* (Kerhoas et al. 2006); however, such reduction might be enough to confer a lighter colouration to *abc1k2* seeds (Fig. 5B**)**.

In Arabidopsis seeds, sinapoylcholine represents the most abundant metabolite derived from sinapic acid (Shirley et al. 2003). In both wild-type and mutant seeds, the content of sinapoylcholine showed no differences between the two genotypes (Supplemental Table 2). In contrast, other sinapic acid derivatives, including the glycosylated forms and those putatively conjugated with glucosinolates, increased in the mutant line (Fig. 5A). The presence of sinapoylated glucosinolates in Arabidopsis seeds (Supplementary Table 2) was deduced by monitoring the presence of characteristic fragment ions in the MS/MS function (Shybyray et al. 2025). Regarding glucosinolates (GLS), non-sinapoylated forms exhibited distinct profiles between wild-type and mutant plants. Specifically, sulfinyl-GLSs were more abundant in the mutant, whereas methylsulfanyl glucosinolates and benzoyloxy moieties were more represented in the wild-type. Interestingly, the levels of the most abundant glucosinolate, *i.e.* glucoerucin (4-methylthiobutyl-GLS), were comparable to those in the wild-type. In contrast, its putative sinapoylated form and the other putative sinapoylated glucosinolates, as tentatively identified in our study, were primarily accumulated in the seeds of the mutant line.

### The loss of *ABC1K2* alters the transcriptome in seeds

**Fig. 6:**
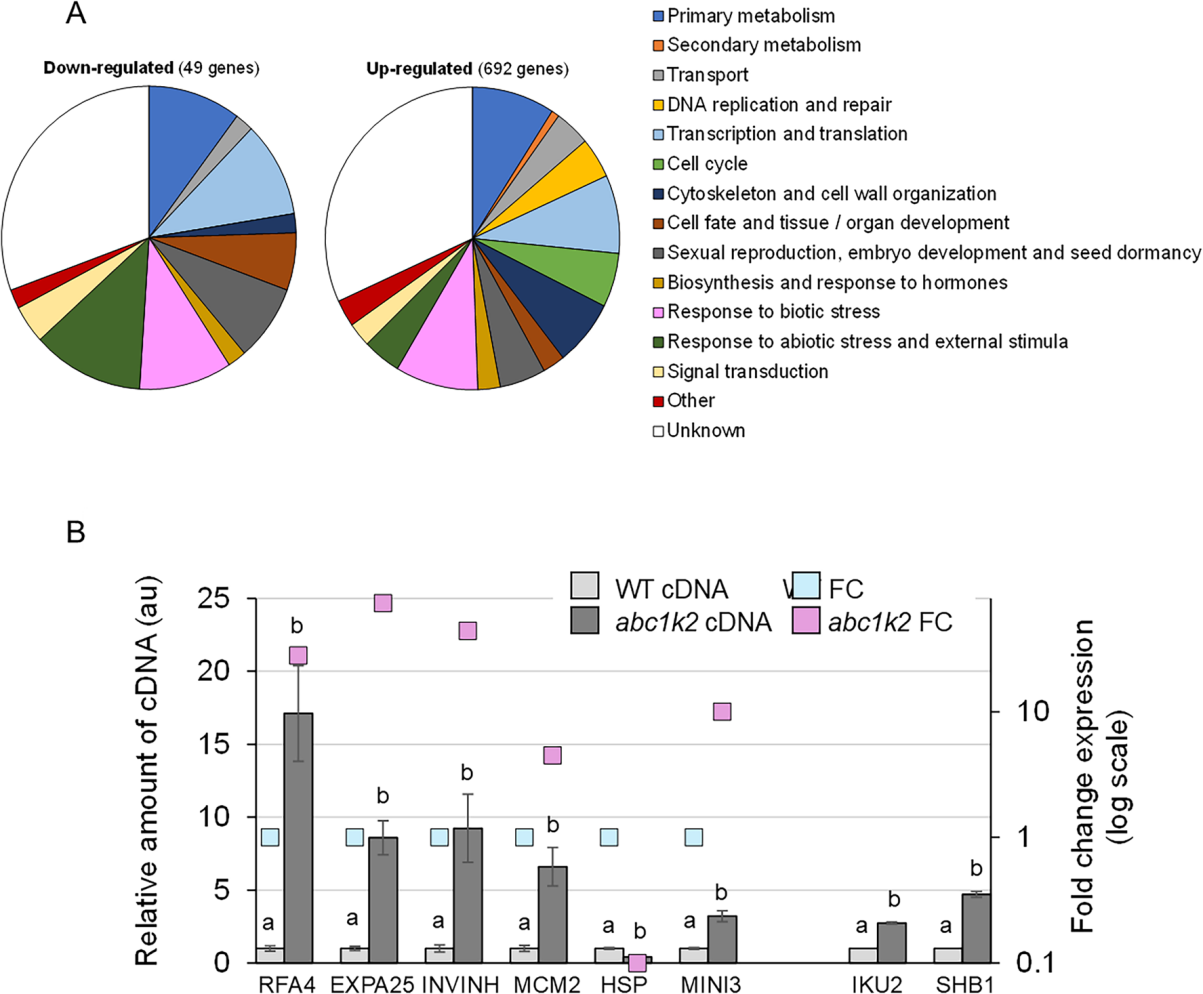
**A**: Functional classification of differentially expressed genes (DEGs) resulting from the microarray transcriptomic comparison between wild-type and *abc1k2* genotypes. The DEGs were classified considering the current annotation as GO terms and the state-of-the-art literature available. **B**: Expression levels of selected differentially expressed genes (DEGs) in the wild-type and abc1k2 siliques. For each genotype, results are reported for both microarray analysis (right Y axis, light blue and purple squares) and real-time RT-PCR (left Y axis, grey histograms) for validation. IKU2 and SHB1 did not emerge as statistically significant DEGs in the transcriptomic analysis, but were analysed as partners of MINI3. Data are reported as mean ± standard error of three biological replicates. Different letters above the bars indicate statistical significance, evaluated by one-way ANOVA followed by a Tukey’s post-hoc test (*p* < 0.05); the statistical analysis was performed independently for each gene analysed, setting as 1 the expression in the wild-type

The transcriptomic analysis comparing wild-type and *abc1k2* seeds produced a total of 741 statistically significant differentially expressed genes (DEGs) with an absolute fold change higher than 2 (Supplementary Table 3). Of these, the majority (692 DEGs) was up-regulated in the *abc1k2* mutant, whereas only 49 genes were down-regulated (Fig. 6A). Functional classification of the identified DEGs highlighted, among the up-regulated sequences, a high occurrence of genes whose function is associable to cell cycle and growth (DNA replication and repair: 4.3%; cell cycle: 5.7%; cytoskeleton and cell wall organization: 7.1%) as well as sexual reproduction, embryo development and seed dormancy (5.1%). Response to abiotic and biotic stresses was also highly represented (abiotic: 4.2% in up-regulated genes, 12.2% in down-regulated; biotic: 9.1% in up-regulated genes, 10.2% in down-regulated) (Fig. 6A). The GO term enrichment analysis performed by DAVID produced no enriched cluster among down-regulated genes. Regarding up-regulated transcripts (Supplementary Table 4), the cluster with the highest score was associated with defence against biotic stresses and included 64 genes described with functions as “antimicrobial” and “fungicide”: indeed, a high occurrence of small cysteine-rich proteins with a putative role in defence was found. However, these proteins are still poorly characterised and may have a variety of functions: in addition to their role in plant-pathogen interaction, they have been proposed to participate in plant reproduction and development, seed storage, signalling and response to environmental cues (Domingo et al. 2024; Pereira Mendes et al. 2021; Silverstein et al. 2007; Xu et al. 2018). In addition to this cluster, the analysis confirmed the enrichment of DEGs involved in cell cycle (clusters 2, 3, 4, 13, 14, 17 and 18), DNA repair (clusters 15 and 19), cytoskeleton and cell wall organization (clusters 6, 10, 21 and 25), seed storage (clusters 5 and 12) and ubiquitin-mediated proteolysis (cluster 27). The high occurrence of modulated genes entering these pathways was confirmed by mapping to the KEGG database (Supplementary Table 5). Modulated genes associated with significant functional clusters and pathways were chosen for real-time RT-PCR validation: the obtained results confirm the trends observed for DEGs in microarray analysis (Fig. 6B). Helicase subunit *MCM2* (*MINICHROMOSOME MAINTENANCE 2*, AT1G44900), as well as five other members of the MCM2-7 complex, are up-regulated in the *abc1k2* mutant; the helicase complex plays an essential role in DNA replication and cell cycle during different stages of embryo and endosperm development (Herridge et al. 2014; Holding and Springer 2002; Ni et al. 2009; Springer et al. 2000). E3 ubiquitin ligase *RFA4* (*RING FINGER ABA-RELATED 4*, AT2G21420), up-regulated in *abc1k2*, mediates degradation of ABA receptors and is, therefore, involved in seed germination and response to abiotic stresses (Fernandez et al. 2020). Down-regulated heat shock proteins *HSP17.6II* (*HEAT SHOCK PROTEIN 17.6II*, AT5G12020) and *HSP17.6A* (AT5G12030) have been associated with tolerance to abiotic stresses (Sun et al. 2001; Yang et al. 2020). Regarding cell wall biosynthesis and organization, expansin *EXPA25* (*EXPANSIN A25*, AT5G39300) and invertase/pectin methylesterase inhibitors *INVINH1* (*INVERTASE INHIBITOR 1*, AT5G46960) and *INVINH2* (AT5G46950) were induced in *abc1k2*; these three genes, involved in the regulation of cell wall expansion, were found as associated with seed and endosperm development (Liu et al. 2020; Zuma et al. 2018). The transcription factor *WRKY10/MINI3* (*MINISEED 3*, AT1G55600) was positively modulated in *abc1k2*; its partners *IKU2* (*HAIKU 2*, AT3G19700) and *SHB1* (*SHORT HYPOCOTYL UNDER BLUE 1*, AT4G25350) did not emerge in the transcriptomic analysis, but their modulation was confirmed as consistent with that of *MINI3* in real-time RT-PCR. The SHB1-MINI3-IKU2 signalling chain is involved in endosperm development and regulates seed size (Luo et al. 2005; Zhou et al. 2009).

## Discussion

### ABC1K2 is expressed in the seeds and localises in granular structures

ABC1K2 is one of the less characterised proteins among the ABC1K kinase family. Although a previously published work included it in the plastidial clade and, by predictive analysis, proposed its localisation in leaves and in the mitochondria (Lundquist et al. 2012), proteomic approaches aiming at identifying the proteome of plastids and plastoglobules have failed to clearly localise ABC1K2 (Lundquist et al. 2013). In this work, *ABC1K2* was found to have a definite expression in the maturing seeds, and in particular in the embryonal tissues. This is the first ABC1K transcript showing an embryo-specific expression; although expression in seeds was observed in rice for *OsAGSW1*, an ortholog of *AtABC1K3* (Li et al. 2015a, b), its expression profile is significantly wider than that observed here for *ABC1K2*. As a result, protein subcellular localisation by proteomic analysis of plastids may have been compromised by tissue distribution: indeed, if the mRNA presence, in standard growth conditions, is detected at great abundance only in forming seeds, proteome analysis of leaf tissue will probably remain inconclusive. The localisation of reporter proteins-tagged ABC1K2 has not been reported so far, and in our hands, the localisation of RFP or GFP-tagged proteins did not give clear results. Nonetheless, we observed fluorescence distributed in small granular structures for both constructs, suggesting a biological significance due to the consistency of the observation. We also observed a weak overlap with plastidial localisation, which was judged not due to residuals from chlorophyll autofluorescence. Moreover, fluorescent fusion proteins may accumulate in peroxisomes, which physically interact with chloroplasts, thus explaining the weak localisation next to chloroplasts (Oikawa et al. 2015). To investigate the nature of the granular distribution, guided by pattern analogy, we co-localised ABC1K2::GFP with the TGN marker ST-RFP and with the mitochondrial MitoTracker Red dye. None of these analyses showed a clear co-localisation, but only partial overlap. In conclusion, even though subcellular localisation was elusive, possibly due to the disturbance of the fluorescent proteins to the ABC1K2 proper folding, the achieved results are suggestive of a possible novel compartment associated with ABC1K proteins.

### ABC1K2 participates in the ABA-mediated response to abiotic stress

Despite being only minimally expressed in vegetative tissues under normal growth conditions, this work highlighted that *ABC1K2* is significantly induced upon ABA and NaCl treatment and that this is mediated by *cis* ABA- and stress-responsive elements in the promoter region. The modulation of ABC1K genes in response to abiotic stress is a well-documented phenomenon (Li et al. 2015a, b; Gao et al. 2022; Chen et al. 2024; Wang et al. 2024). More in detail, some ABC1Ks, such as Arabidopsis ABC1K7/AtSIA and ABC1K10a, and cotton GhABC1K2-A05 and GhABC1K12-A07, have been implicated in the tolerance to salt stress, mainly affecting ROS production and the energy chain (Yang et al. 2012a, b; Qin et al. 2020; Wang et al. 2024). Similarly, ABC1Ks have also been reported as involved in ABA signalling, as, for example, ABC1K7 and ABC1K8 in Arabidopsis (Manara et al. 2016). Interestingly, knock-out mutations of the latter two genes, both belonging to the same plastidial clade as ABC1K2, produced analogous changes in ABA-mediated processes, similarly resulting in ABA insensitivity related to stomatal closure and altered ABA-induced senescence (Manara et al. 2016). Overall, ABC1K2 participates in the modulation of abiotic stress responses, particularly mediated by ABA, in a pattern that is common in the ABC1K kinase family; however, *abc1k2* plants do not show the typical phenotype of ABA-deficient mutants, as those shown by *aba2-1*, such as shorter siliques, early flowering and growth retardation (Cheng et al. 2014). In the case of ABC1K2, the gene is not highly transcribed in somatic or vegetative tissues in the absence of stress, but only in seeds during embryogenesis. Therefore, its role in vegetative tissues is likely limited to stress response due to the extremely specific expression pattern displayed in normal conditions. Overall, these results are consistent with a reduced response of the mutant to ABA signalling, which, however, does not compromise plant fitness and reproduction under standard, controlled growth conditions.

### ABC1K2 participates in the coordination of seed development

As mentioned above, ABC1K2 seed-specific localisation is a novelty in the context of the ABC1K kinase family. Although an involvement of the ABC1K family in seed development was already postulated for rice OsAGSW1, the latter seems to be involved in different processes than ABC1K2 (Li et al. 2015a, b). Consistent with the observed expression pattern, *ABC1K2* loss of function impacts seed development, secondary metabolism and germination. These differences, although not severe enough to impair plantlet vitality or fitness, still mark ABC1K2 as a potential core modulator connecting different aspects of seed development.

First of all, *abc1k2* germinated earlier when in the after-ripening condition. This evidence correlated with a significantly lower ABA content in mature, freshly harvested seeds, and indeed ABA is for sure the key determinant of dormancy setting during seed development (Bewley 1997; Koorneef et al. 2002). In our case, *ABC1K2* loss of function does not impair seed dormancy, and indeed the *abc1k2* mutant did not exhibit the most extreme ABA-related phenotypes such as non-dormant seeds (Nambara et al. 1992). On the other hand, it has been demonstrated that ABA content does not change from freshly harvested to after-ripening seeds, even if seed conservation alters the rate of seed germination after imbibition (Millar et al. 2006). In this context, while the ABA content of both wild-type and *abc1k2* seeds was enough to maintain dormancy in freshly harvested seeds, our storage conditions were sufficient to release dormancy only in the mutant line, likely due to the lower ABA content. Seed dormancy has been shown to be defined early during seed development, by the concurrent action of different signalling cascades having the balance of gibberellins and ABA as core components (reviewed by Holdsworth et al. 2008; Verma et al. 2022). Interestingly, among the genes up-regulated in *abc1k2*, *LEC1* (*LEAFY COTYLEDON 1*) and *LEC2* belong to the core mechanism controlling seed maturation, including the acquisition of dormancy (Roscoe et al. 2015; Bryant et al. 2019).

In addition to the changes observed in germinability, *abc1k2* seeds were bigger than wild-type. Consistently, ABA signalling is also involved in the determination of seed dimensions; indeed, loss of function in ABA biosynthesis (*aba2-1* mutant), translocation (*mir17,18* knock-down lines) and ABA-mediated transcription regulation (*abi5* mutant) produced increased seed size (Cheng et al. 2014; Zhang et al. 2025), similarly to *abc1k2*. ABA2 and ABI5 have been demonstrated to modulate seed size by regulating the IKU pathway (Cheng et al. 2014). The SHB1-MINI3-IKU2 signalling cascade has been reported to regulate seed size by controlling both embryo and endosperm development (Garcia et al. 2003; Luo et al. 2005; Zhou et al. 2009). Notably, all three genes were up-regulated in *abc1k2*, although no differences in transcription levels emerged regarding the ABA-related transcription factors upstream of their signalling chain; however, in the latter, regulation may occur through the management of protein activity rather than transcription, and therefore be missed in the transcriptomic analysis.

The network controlling seed size and development is extremely complex and involves several distinct pathways (reviewed by Orozco-Arroyo et al. 2015; Li and Li 2016). Consistent with this complex scenario, *abc1k2* mutants showed up-regulation in a wide variety of genes that participate in DNA replication and repair, cell division, cell wall biosynthesis and modification, as well as in the ubiquitin–proteasome pathways; the latter has been directly implicated in the control of seed size (Li and Li 2016). Overall, the changes in the *abc1k2* transcriptome reflect an alteration of the normal seed metabolism, which, although not detrimental for the plant fitness, is enough to bring about evident phenotypical changes in the seed development.

### ABC1K2 modulates secondary metabolism in seeds

In addition to the previously discussed changes observed in seed maturation, the *abc1k2* mutant showed a significant alteration in seed secondary metabolism. In particular, moderate- to high-confidence identification of candidate metabolites revealed consistent changes across the classes of glucosinolates and phenylpropanoids. Glucosinolates (GLSs) are nitrogen- and sulfur-containing secondary metabolites, derived mostly from methionine and tryptophan, that play a role in protecting plants from micro-herbivores and, in some species, are involved in auxin biosynthesis (Lee et al. 2012). 2-Phenylethyl glucosinolate and compounds with benzoyloxy substituents were identified as most abundant in *Arabidopsis* seeds (Brown et al. 2003). In *abc1k2* seeds, some GLS moieties that had been identified as typical of the seed tissues, such as 7-methylthioheptyl-, 8-methylthiooctyl-, 3-benzoyloxypropyl- and 4-benzoyloxybutyl- GLSs (Brown et al. 2003), showed a reduced accumulation. Globally, while benzoylated moieties appeared much less abundant in *abc1k2* seeds compared to the wild-type, the contrary was observed for the putatively sinapoylated compounds, especially 4-methylthiobutyl-, 8-methylsulfinyloctyl-, 7-methylsulfinylheptyl- GLS, with the latter not previously identified as sinapoylated (Lee et al. 2012). Interestingly, since GLS biosynthesis has been reported as being mainly under maternal control (Nour-Eldin and Halkier 2009; Xu et al. 2023), sinapoylation might occur in the embryo on GLSs that are imported from maternal tissues. Consistently, *abc1k2* showed significantly higher expression levels of two serine carboxypeptidase-like (SCPL) acyltransferases, *SCPL21* and *SCPL47*: although not well characterised, they belong to a class of proteins that have been directly linked with sinapoylation of specific substrates, including GLSs (Lee et al. 2012). Moreover, it has long been known that sinapoylation of choline from sinapoylglucose occurs in seeds, and thus, active donors of sinapic acid are at least available in seed tissue (Nour-Eldin et al. 2012). In *abc1k2* seeds, sinapoylglucose accumulation was enhanced, possibly fuelled by an enhanced sinapate biosynthetic pathway. Notably, this metabolic change seems to have the most effect on GLS modification, rather than on the synthesis of sinapoylcholine, whose content was not changed in the mutant, or of sinapoylmalate, which has not been identified in the seeds of either genotype. Since sinapic acid esters are involved in protection against UV radiation, in seed germination and seedling development in Brassicaceae (Milkowski and Strack 2010), we propose that the enhanced content of putative sinapoylated esters in *abc1k2* may play a role in its enhanced germination. Consistently, it has been recently shown that treatment of wild-type seeds with sinapic acid enhances seed accumulation of sinapoylglucose and, in turn, seed germination (Bi et al. 2017). Interestingly, the pathways associated with ABA and sinapates in seeds seem to be connected. As previously discussed, ABA content in *abc1k2* seeds was reduced, and this could also be tentatively ascribed to the enhanced accumulation of sinapoylated esters, similarly to what happens upon chemical treatment of seeds with sinapic acid (Bi et al. 2017).

GLS sinapoylation also likely influenced the accumulation of phenylpropanoids in *abc1k2*. While hydroxycinnamic acids, as a class and in particular the sinapic acid derivatives, were more abundant in the mutant, phenylpropanoids were mostly underrepresented in *abc1k2* in comparison with the wild-type. This can be explained as a reorganisation of *abc1k2* secondary metabolism, which probably favours the consumption of coumaric acid and other hydroxycinnamic acids into the pathway of sinapic acid production, to be potentially loaded in great amounts into GLS biosynthesis and modification. Moreover, GLS and phenylpropanoid biosynthetic pathways have been proposed to interact in their early stages, leading to mutual modulation (Kim et al. 2015). As a result, flavonols and proanthocyanidins were less abundant upon *ABC1K2* loss of function. Notably, higher phenylpropanoid contents, particularly proanthocyanidins, have been correlated with lower seed coat permeability and ABA de novo biosynthesis (Jia et al. 2012; MacGregor et al. 2015), thus further explaining the stronger dormancy of wild-type seeds in comparison to *abc1k2*.

## Concluding remarks

The functional characterisation of ABC1K proteins is still an ongoing process, with many members that are the subject of current investigations. ABC1K2 is one of these, with no shared high sequence similarity (and therefore, functional redundancy) with other members of this gene family. Under standard growth conditions, *abc1k2* mutants manage to complete their life cycle, setting up vital seeds, showing that the lack of ABC1K2 is not detrimental to plant life. On the other hand, it seems involved in processes that regulate ABA content and response, and secondary metabolites accumulation in the seed. Even though further studies will be necessary to unravel the protein's subcellular localisation, our study gives hints that ABC1K2 may play a role in the fine tuning of the network between ABA, abiotic stress response, secondary metabolism and seed development.

## Supplementary Information

Below is the link to the electronic supplementary material.

Supplementary file1 (TIF 199 KB)

Supplementary file2 (TIF 2369 KB)

Supplementary file3 (TIF 254 KB)

Supplementary file4 (TIF 257 KB)

Supplementary Table 1: Detailed table of transcription factor binding sites identified in the ABC1K2 promoter sequence by the PlantPan 4.0 algorithm. Supplementary file5 (XLSX 57 KB)

Supplementary Table 2: Results of the comparative analysis of the seed secondary metabolites by LC-HRMS analysis by an untargeted metabolomics approach in negative ionisation mode. Putatively identified signals were grouped in specific chemical classes and compared between wild-type and abc1k2 mutant. Supplementary file6 (XLSX 86 KB)

Supplementary Table 3: Transcriptomic analysis in siliques of wild-type and abc1k2 plants. A total of 741 statistically significantly differentially expressed genes (DEGs) with an absolute fold change higher than 2 are listed and grouped into up-regulated and down-regulated in abc1k2. Supplementary file7 (XLSX 152 KB)

Supplementary Table 4: DAVID functional annotation on up-regulated genes identified by the transcriptomic analysis. Supplementary file8 (XLSX 47 KB)

Supplementary Table 5: List of association categories of modulated genes based on known metabolic pathways in Arabidopsis by using the KEGG Mapper tool in the KEGG database. Supplementary file9 (XLSX 18 KB)

## Data Availability

All relevant data can be found within the manuscript and its supporting materials. Raw transcriptomic data are deposited at GEO (accession nr. GSE299982).
