# Supplementary figures and images for: ABC1K2 is involved in stress response and secondary metabolism during seed development in Arabidopsis thaliana
Source: Plant Cell Rep. 2025 Oct 29;44(11):249. doi: 10.1007/s00299-025-03645-0 (PMC12568898; doi:10.1007/s00299-025-03645-0)

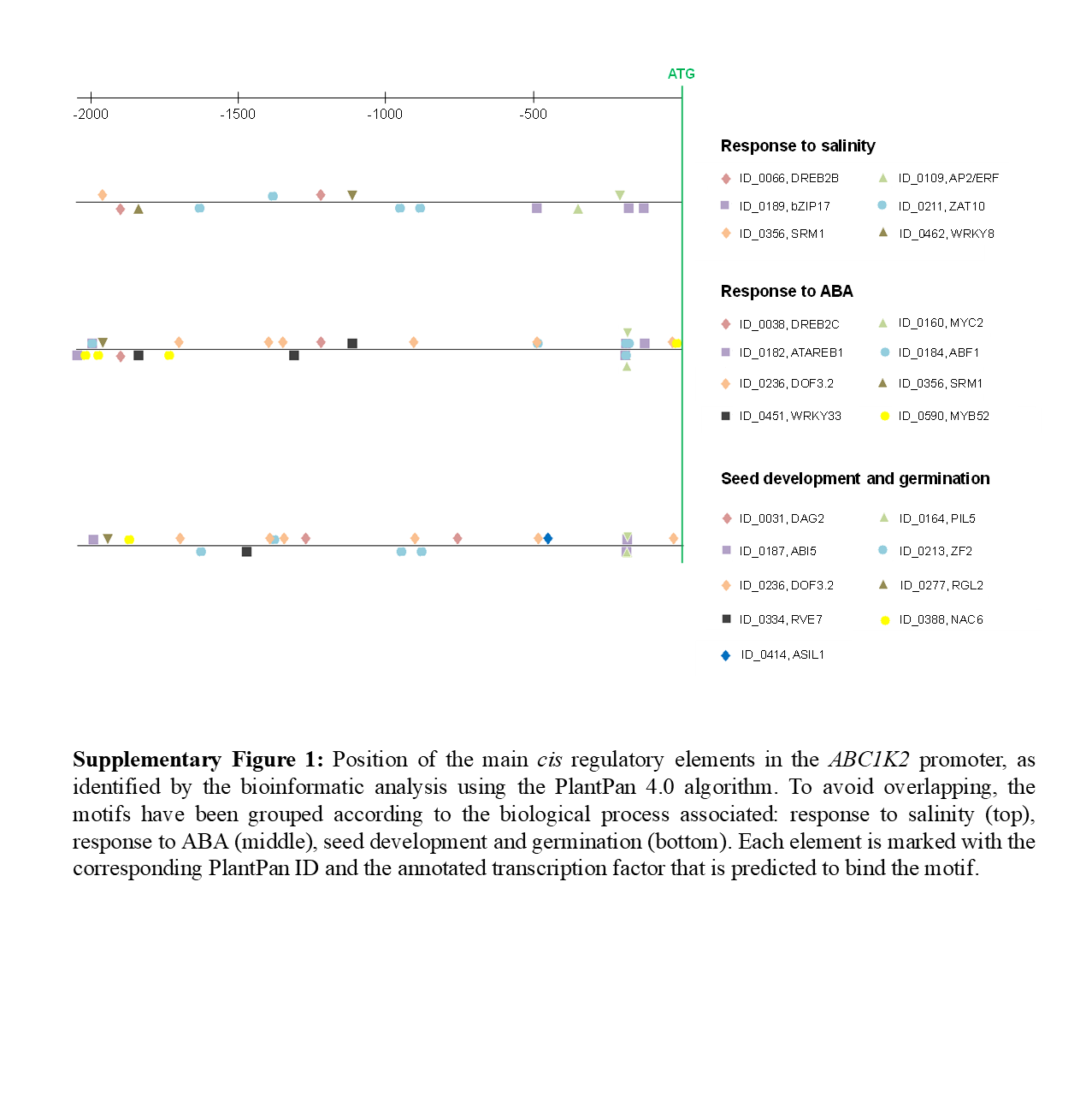

Supplement: Supplementary file 1 — Supplementary file1 (TIF 199 KB) [file 299_2025_3645_MOESM1_ESM.tif]

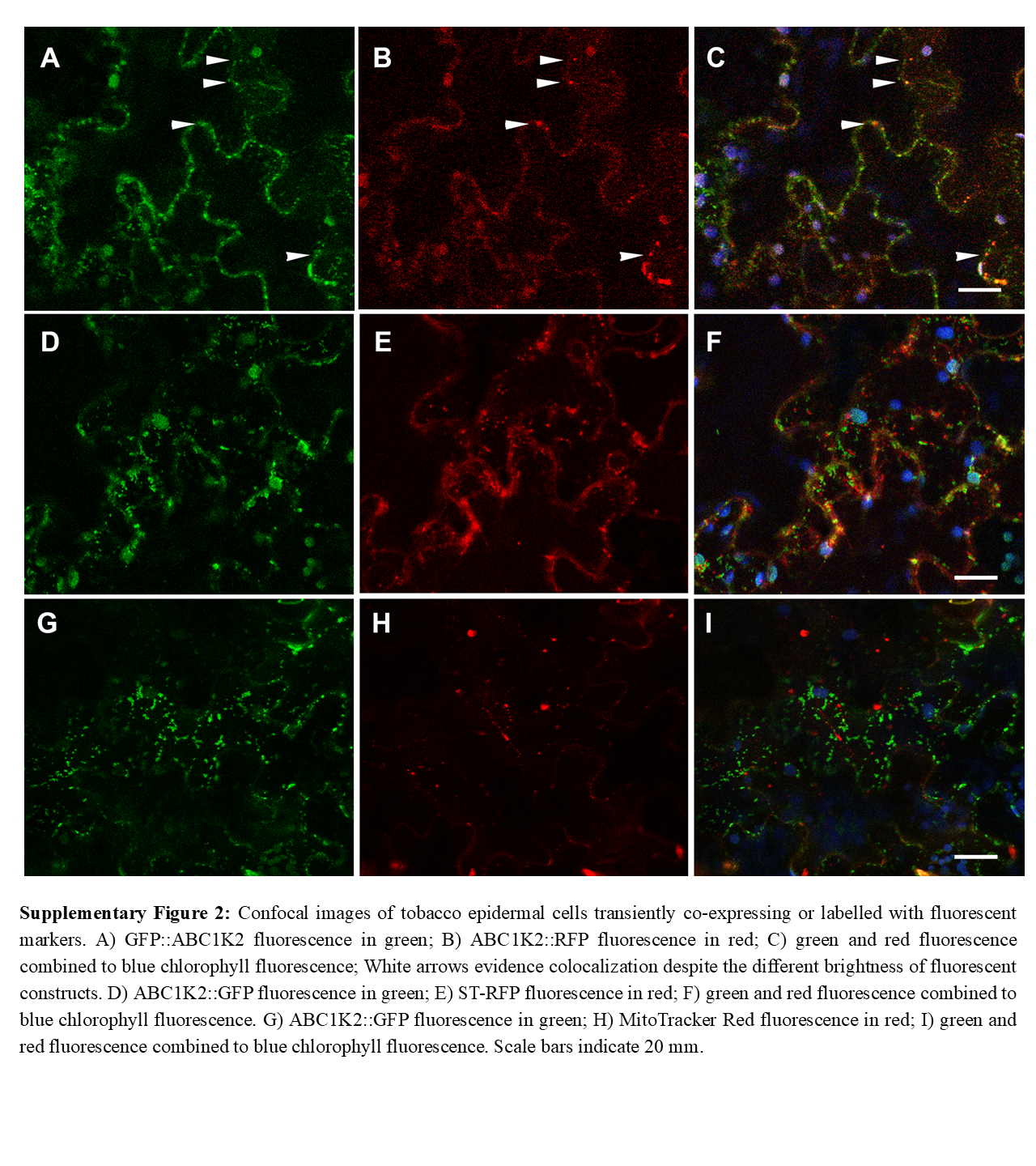

Supplement: Supplementary file 2 — Supplementary file2 (TIF 2369 KB) [file 299_2025_3645_MOESM2_ESM.tif]

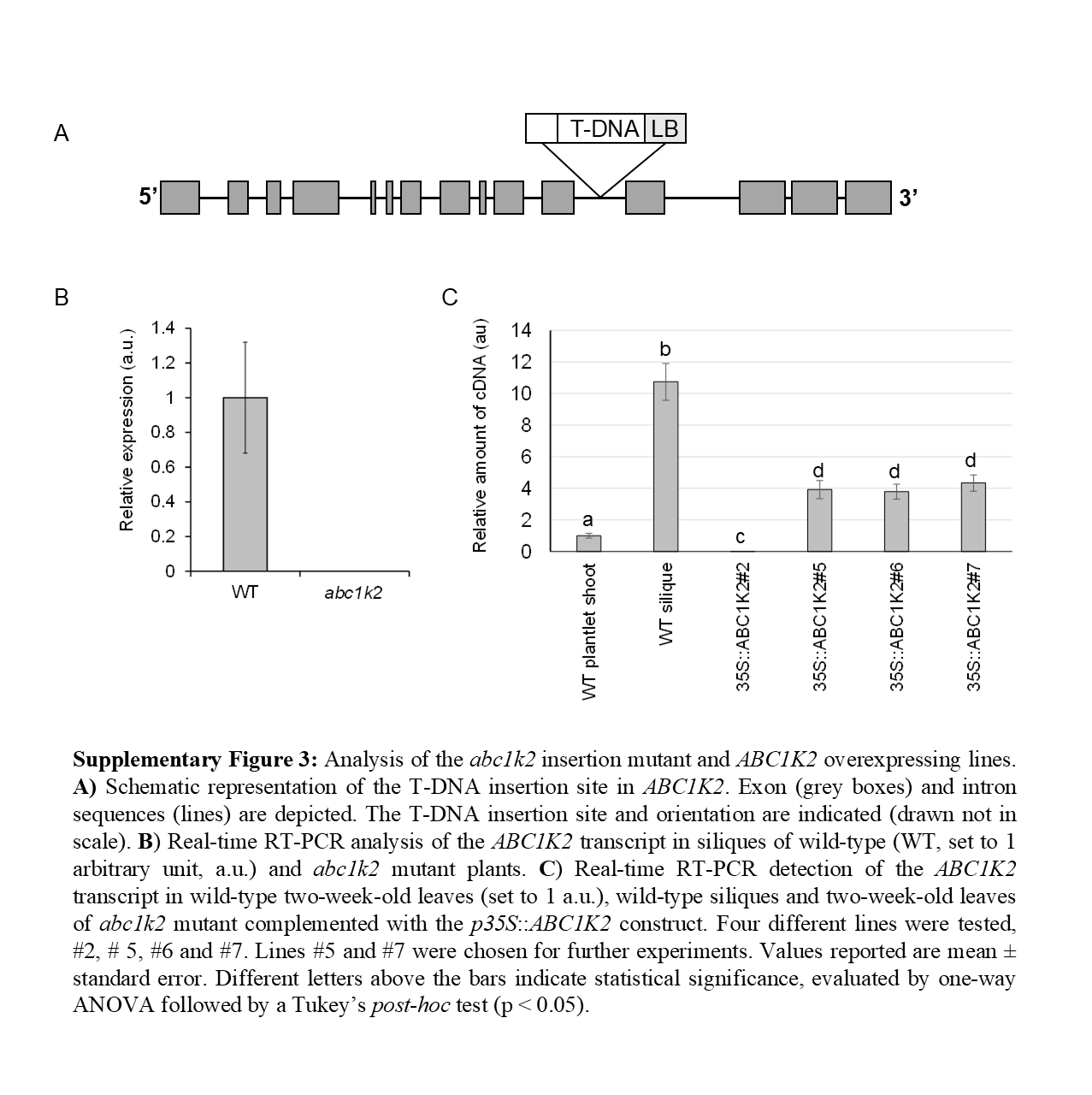

Supplement: Supplementary file 3 — Supplementary file3 (TIF 254 KB) [file 299_2025_3645_MOESM3_ESM.tif]

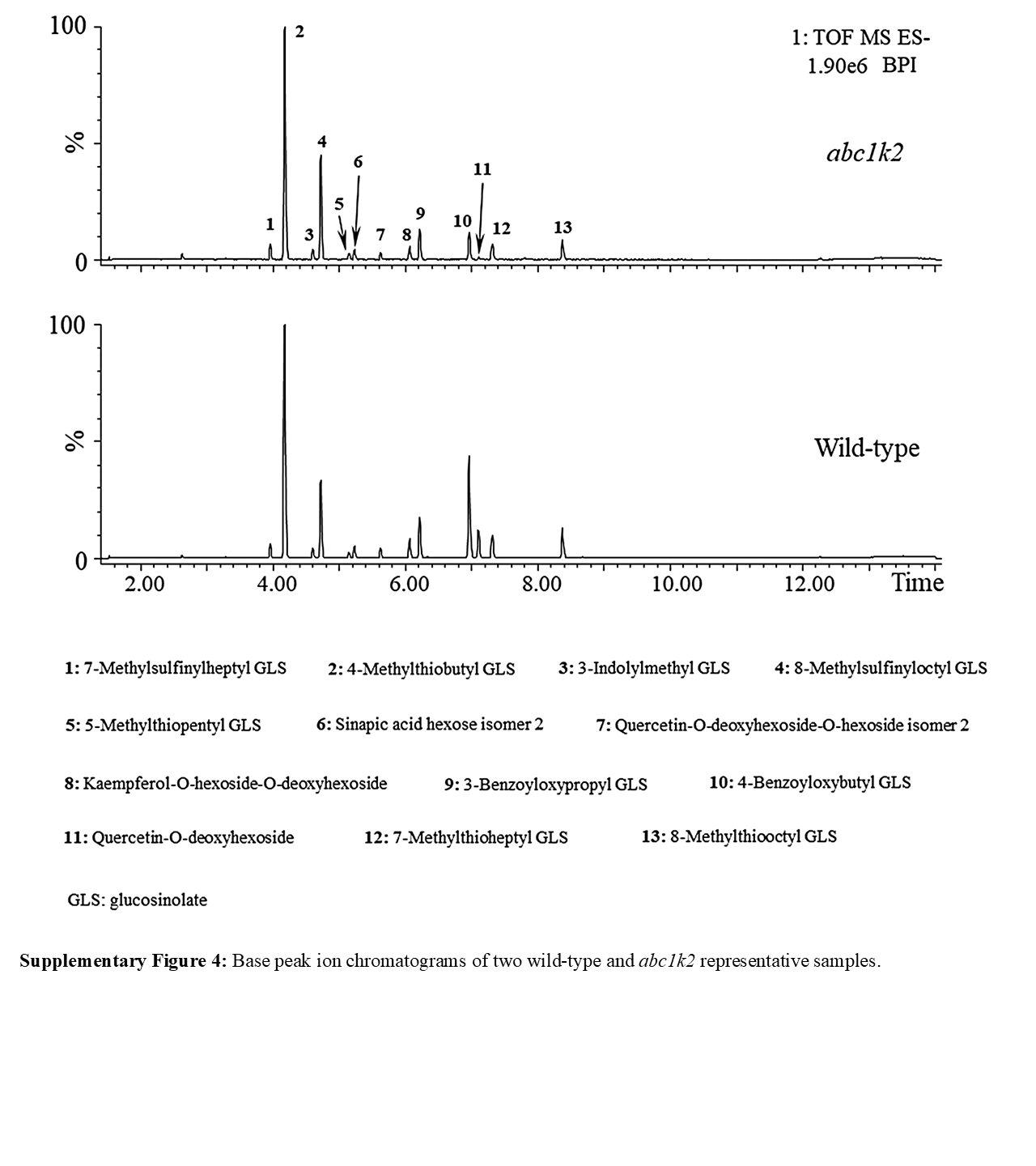

Supplement: Supplementary file 4 — Supplementary file4 (TIF 257 KB) [file 299_2025_3645_MOESM4_ESM.tif]
